# Natural Horizontal Gene Transfer of Antimicrobial Resistance Genes in *Campylobacter* spp. From Turkeys and Swine

**DOI:** 10.3389/fmicb.2021.732969

**Published:** 2021-09-27

**Authors:** Vanina Guernier-Cambert, Julian Trachsel, Joel Maki, Jing Qi, Matthew J. Sylte, Zahra Hanafy, Sophia Kathariou, Torey Looft

**Affiliations:** ^1^Food Safety and Enteric Pathogens Research Unit, United States Department of Agriculture, Agricultural Research Services, National Animal Disease Center, Ames, Ames, IA, United States; ^2^Agricultural Research Service Research Participation Program, Oak Ridge Institute for Science and Education, Oak Ridge, TN, United States; ^3^Interdepartmental Microbiology Graduate Program, Iowa State University, Ames, IA, United States; ^4^Shandong Academy of Agricultural Sciences, Institute of Animal Science and Veterinary Medicine, Jinan, China; ^5^Department of Food, Bioprocessing and Nutrition Sciences, North Carolina State University, Raleigh, NC, United States

**Keywords:** antimicrobial resistance, *Campylobacter*, transformation, turkey, swine, animal experiment, co-culture experiment, horizontal gene transfer

## Abstract

Antibiotic-resistant *Campylobacter* constitutes a serious threat to public health. The clonal expansion of resistant strains and/or the horizontal spread of resistance genes to other strains and species can hinder the clinical effectiveness of antibiotics to treat severe campylobacteriosis. Still, gaps exist in our understanding of the risks of acquisition and spread of antibiotic resistance in *Campylobacter*. While the *in vitro* transfer of antimicrobial resistance genes between *Campylobacter* species *via* natural transformation has been extensively demonstrated, experimental studies have favored the use of naked DNA to obtain transformants. In this study, we used experimental designs closer to real-world conditions to evaluate the possible transfer of antimicrobial resistance genes between *Campylobacter* strains of the same or different species (*Campylobacter coli* or *Campylobacter jejuni*) and originating from different animal hosts (swine or turkeys). This was evaluated *in vitro* through co-culture experiments and *in vivo* with dual-strain inoculation of turkeys, followed by whole genome sequencing of parental and newly emerged strains. *In vitro*, we observed four independent horizontal gene transfer events leading to the acquisition of resistance to beta-lactams (*blaOXA*), aminoglycosides [*aph(2′′)-If* and *rpsL*] and tetracycline [*tet(O)*]. Observed events involved the displacement of resistance-associated genes by a mutated version, or the acquisition of genomic islands harboring a resistance determinant by homologous recombination; we did not detect the transfer of resistance-carrying plasmids even though they were present in some strains. *In vivo*, we recovered a newly emerged strain with dual-resistance pattern and identified the replacement of an existing non-functional *tet(O)* by a functional *tet(O)* in the recipient strain. Whole genome comparisons allowed characterization of the events involved in the horizontal spread of resistance genes between *Campylobacter* following *in vitro* co-culture and *in vivo* dual inoculation. Our study also highlights the potential for antimicrobial resistance transfer across *Campylobacter* species originating from turkeys and swine, which may have implications for farms hosting both species in close proximity.

## Introduction

With an estimated 9.4 million yearly cases in the United States alone, human foodborne diseases are an important public health concern (Scallan et al., 2011). Among bacterial foodborne pathogens, *Campylobacter* and *Salmonella*, cause the largest proportion of bacterial illnesses in the United States and other industrialized nations. In 2019, among the eight bacterial pathogens monitored by the Foodborne Diseases Active Surveillance Network (FoodNet), the overall incidence per 100,000 population was highest for *Campylobacter* (19.5 cases; Tack et al., 2020). While campylobacteriosis is largely a self-limiting illness, it is accompanied by acute gastroenteritis and is a leading antecedent for the severe autoimmune complication Guillain-Barré syndrome (Allos, 1997; Nachamkin, 2002; Scallan Walter et al., 2020). This, together with additional autoimmune sequelae, such as post-infectious irritable bowel syndrome and reactive arthritis, confers a high disease burden to human campylobacteriosis in the United States and worldwide (Batz et al., 2013; Kirk et al., 2015; Scallan et al., 2015; Scallan Walter et al., 2019).

*Campylobacter* spp. are Gram-negative bacteria that colonize the gastrointestinal tract of various animals. Since its first description in 1963, the genus has grown to include several important human and animal pathogens (Man, 2011). Two species, *Campylobacter coli* and *Campylobacter jejuni*, are the primary contributors to human campylobacteriosis (>Nachamkin et al., 2008). *Campylobacter* gastroenteritis usually occurs after contact with raw poultry, ingestion of undercooked animal products, raw milk/vegetables/fruits, or contaminated water, or direct transmission from pets and other animals (Facciola et al., 2017). Poultry are recognized as the most important animal reservoir for infection to humans, with 60–80% of human cases in Europe attributable to exposure to raw or undercooked poultry products [European Food Safety Authority (EFSA) and European Centre for Disease Prevention and Control (ECDC), 2015].

Ciprofloxacin (fluoroquinolone) and azithromycin (macrolide) are the drugs of choice to treat *Campylobacter* enteritis, while intravenous aminoglycosides are used for serious bacteremia and other systemic infections due to *Campylobacter* (Aarestrup and Engberg, 2001). Recent years have seen a worldwide rise in *Campylobacter* antimicrobial resistance (AMR) to fluoroquinolones (Gupta et al., 2004; Nelson et al., 2007) and, to a lesser extent, macrolides and other classes of antibiotics (Padungton and Kaneene, 2003; Moore et al., 2006; Luangtongkum et al., 2009). As *Campylobacter* spp. are zoonotic pathogens, the development and spread of AMR in the animal reservoir may significantly limit the options for clinical treatment of campylobacteriosis in humans (Moore et al., 2006). Every year, an estimated 700,000 deaths are attributable to antimicrobial-resistant infections worldwide, a number projected to rise to 10 million deaths per year by 2050 in the absence of additional control measures (O’Neill, 2014). In 2019, the CDC listed drug-resistant *Campylobacter* as a serious threat, causing twice as many infections (448,400) in the United States each year as drug-resistant *Salmonella* [Centers for Disease Control and Prevention (CDC), 2019], and the World Health Organization has listed fluoroquinolone-resistant *Campylobacter* as one of six high priority antimicrobial-resistant bacteria (Tacconelli et al., 2018).

Several studies have linked the use of antimicrobials – in particular fluoroquinolones – in food animal production and veterinary medicine with the emergence and spread of resistant *Campylobacter* (Alfredson and Korolik, 2007). In the United States, the introduction of the fluoroquinolones, sarafloxacin, and enrofloxacin in the 1990s as growth promoters in poultry has been associated with an increase of fluoroquinolone-resistant *Campylobacter* in human cases (Anderson et al., 2003).

*Campylobacter* spp. are intestinal commensals shared among livestock species, thus providing a possible pathway for AMR to spread between different host species. *Campylobacter jejuni* is the dominant *Campylobacter* species in poultry while *C. coli* is the most common species recovered from swine (Horrocks et al., 2009), but this host specificity is not strict. A study conducted in China between 2008 and 2014 observed an apparent shift of the dominant species from *C. jejuni* to *C. coli* in chickens, with this species shift coinciding with an increased prevalence of macrolide-resistant *C. coli* (Wang et al., 2016). Interestingly, both species colonize turkeys at high rates (Wright et al., 2008; Wesley et al., 2009).

The *in vitro* transfer of antimicrobial resistance genes (ARGs) between different *Campylobacter* species *via* natural transformation has been extensively demonstrated. For instance, *erm(B)* and *aph(2″)-If* (conferring resistance to macrolides and aminoglycosides, respectively) could be transferred from *C. coli* to *C. jejuni via* natural transformation (Qin et al., 2014; Wang et al., 2014). However, previous experimental studies have favored the use of extracted DNA in transformations. The aim of this study was to evaluate the possible transfer of ARGs between different *Campylobacter* strains of the same or different species (*C. coli* and *C. jejuni*) and originating from different host species (swine and turkeys) using experimental designs closer to real-world conditions. This was evaluated both *in vitro*, using co-culture experiments, and *in vivo*, using experimental dual-strain infections of turkeys.

## Materials and Methods

### *Campylobacter* Strains and Growth Conditions

The *C. jejuni* and *C. coli* strains used in this study were isolated from turkey feces or ceca, from swine feces, or from the environment at different farms in eastern North Carolina, South Carolina, or Virginia between 2003 and 2014 (Table 1). Strains were first isolated using direct plating on modified charcoal cefoperazone deoxycholate agar (mCCDA; Oxoid, Basingstoke, Hampshire, England) and incubated in anaerobic jars containing a CampyPak Plus microaerobic system (Becton Dickinson, Sparks, MD) at 42°C for 48h (Smith et al., 2004). Strains were then subcultured on different media: (i) non-selective Mueller-Hinton agar (MHA; Becton Dickinson, United States), (ii) modified selective Campy Line agar (CLA-S; Line, 2001) containing 25μg/ml sulfamethoxazole in addition to the original antibiotics (trimethoprim, vancomycin, and polymyxin B; Line et al., 2008), and (iii) selective and differential CHROMagar^™^ Campylobacter (CAC; CHROMagar, Paris, France), and incubated at 42°C in an 10% CO_2_ incubator.

**Table 1:** *Campylobacter jejuni* and *Campylobacter coli* strains used in this study.

Species and strain^1^	Sample	Source	Sampling date
Cc 6461	feces	swine	04/2004
Cc 6067	drinker water^*^	turkey house	11/2003
Cc 13150	feces	turkey	09/2012
Cj 15152A	cecum	turkey	11/2004
Cj 15152B	cecum	turkey	11/2004
Cj 14229–5	feces	turkey	08/2013
Cj 14398–5	feces	turkey	09/2013
Cj 11601MD	jejunum	turkey	09/2006
Cj 6631	feces	turkey	05/2004
Cj 6535	feces	turkey	04/2004
Cj JCC	cecum	turkey (processing plant)	10/2014

1 Strains highlighted in gray have been sequenced previously (Dutta et al., 2016).

* Water sampled from the drinker in the turkey house.

Cc, *Campylobacter coli* and Cj, *Campylobacter jejuni*.

### Antibiotic Susceptibility Profiles

*Campylobacter* spp. strains were selected on the basis of their AMR profiles. They were initially tested on MHA plates with selected antibiotics at the following concentrations: gentamicin (50μg/ml), kanamycin (50μg/ml), streptomycin (50μg/ml), ampicillin (50μg/ml), erythromycin (20μg/ml), and tetracycline (15μg/ml). The same concentrations of antibiotics were used in selective MHA used to recover “newly emerged strains” (see next paragraph) after *in vitro* and *in vivo* experiments. Antibiotics were purchased from Sigma-Aldrich (Saint Louis, Missouri, United States). Selected strains and “newly emerged strains” (see experiments below) were further tested by the Veterinary Diagnostic Laboratory at Iowa State University (ISUVDL) using CAMPY2 Sensititre^®^ plates (VersaTREK Diagnostics/Thermo Fisher, United States) to determine minimal inhibitory concentrations to azithromycin (AZI), ciprofloxacin (CIP), clindamycin (CLI), erythromycin (ERY), florfenicol (FFN), gentamicin (GEN), nalidixic acid (NAL), telithromycin (TEL), and tetracycline (TET) as per the manufacturer’s guidelines. Antimicrobial resistance was determined according to the interpretative criteria recommended by the Clinical and Laboratory Standards Institute [Clinical and Laboratory Standards Institute (CLSI), 2017].

### *In vitro* Transfer of ARGs

*In vitro* experiments were conducted with 11 strain pairs to investigate the possible transfer of ARGs conferring resistance to erythromycin, kanamycin, gentamicin, streptomycin, and tetracycline. Experiments involved a single species (Two pairs with *C. coli*×*C. coli*; 5 pairs with *C. jejuni*×*C. jejuni*) or two species (Four pairs with *C. coli*×*C. jejuni*) and utilized two strains with mutually exclusive resistance for selection. The donor and the recipient strains were identified *a posteriori*. *Campylobacter* colonies showing a dual-resistance pattern, i.e., resistance markers of both parental strains, are hereafter referred to as newly emerged strains (NES) for “newly emerged strain.”

Whole genome sequencing was performed to identify if the newly acquired phenotypic resistance was due to a resistance-associated point mutation, a displacement of a preexisting non-functional resistance gene by a functional version, or a new acquisition of a resistance gene. The experiments utilized three different techniques:

#### Transformation With Naked DNA on Solid Media

Donor DNA was extracted from a *Campylobacter* liquid culture grown in Mueller-Hinton broth using the PureLink^™^ Genomic DNA kit (Invitrogen, Carlsbad, CA) following the manufacturer’s instructions. A loopful from a 24-h-old culture of one strain grown on CLA-S microaerobically at 42°C was spotted into MHA plates (five spots per plate) and 4μl genomic DNA (80μg/μl) of the other strain (this was only tested with strain 6461 DNA) was added to each spot and mixed, with the diameter of each spot after mixing being *ca*. 0.5cm. The plates were incubated up to 24h microaerobically at 42°C.

#### Co-culture of Two Strains on Solid and Biphasic Media

A loopful of the two strains was spotted into MHA solid media (five spots per plate) on the same spot and mixed, with the diameter of each spot after mixing being *ca*. 0.5cm. The plates were incubated for up to 24h microaerobically at 42°C. The biphasic medium consisted of 5ml of MHA overlaid with 5ml of Mueller-Hinton broth in a sterile vented CELLSTAR^®^ 25-cm^2^ tissue culture flask (Greiner Bio-One North America Inc., United States). A loopful of both strains was added to the media in duplicate and co-cultured for up to 24h microaerobically at 42°C. After incubation, either all of the material from a spot (solid media) or 100μl of broth (biphasic media) was spread on 12-cm-diameter MHA plates containing either individual antibiotics (for the recovery and enumeration of parental strains) or an appropriate combination of antibiotics (for the recovery and enumeration of NES). This added up to three selective plates per replicate, for a total of seven replicates (five spots from solid media and two replicates from biphasic media). Colonies were further plated on selective CAC to confirm *Campylobacter*.

### Experimental Design of the Animal Study

This experiment was conducted according to National Animal Disease Center Institutional Animal Care and Use Committee approved protocol. Male day-of-hatch Nicolas turkey poults (*n*=135) were obtained from a commercial hatchery and co-housed upon delivery and the following day to acclimate prior to the start of the study. The rooms were on a 12-h light/dark cycle with positive air pressure (> 10 air changes per hour). Turkeys were fed a turkey starter ration (Damron and Sloan, 1995) *ad libitum* for the entire 6-week study. After the acclimation, poults were randomly split into four rooms: three rooms (*n*=35 each) for inoculated birds and one (*n*=30) for controls. In the control group, (*n*=30) birds were gavaged with 1ml of sterile 1×phosphate-buffered saline (PBS) at Day 2 and euthanized for necropsy in groups of 10 on Days 8, 15, and 22 (Table 2). In the inoculation groups, birds were inoculated by oral gavage with 1ml of a *Campylobacter* suspension at Day 2 and a subset (*n*=10 per room) was euthanized for necropsy on Day 8. The remaining birds (*n*=25) were inoculated at Day 9 (with a strain different to the one inoculated at Day 2) with some (*n*=10) euthanized for necropsy on Day 15 and the remaining birds (*n*=15) euthanized for necropsy on Day 22 (Table 2). Birds were euthanized by CO_2_ asphyxiation followed by cervical dislocation and the cecal contents of each poult were harvested and placed in 50-ml tubes which were stored on ice until analysis for *Campylobacter*, typically within 2h.

**Table 2:** Design and timeline of turkey infections.

Bird age (days)	Day^1^	Room 7 (control group)	Room 9 (inoculation group)	Room 10 (inoculation group)	Room 11 (inoculation group)
Hatch	D0	delivery/co-mingling (*n*=135)		–	–
1	D1	co-mingling (*n*=135)		–	–
2	D2	*n*=30 PBS	*n*=35 Inoculation Cc6461	*n*=35 Inoculation Cc13150	*n*=35 Inoculation Cc6067
8	D8	Necropsy *n*=10	Necropsy *n*=10	Necropsy *n*=10	Necropsy *n*=10
9	D9	–	*n*=25 Inoculation Cj14229-5	*n*=25 Inoculation Cc6461	*n*=25 Inoculation Cc6461
15	D15	Necropsy *n*=10	Necropsy *n*=10	Necropsy *n*=10	Necropsy *n*=10
22	D22	Necropsy *n*=10	Necropsy *n*=15	Necropsy *n*=15	Necropsy *n*=15

1 Day pertains to experimental time points corresponding to the indicated bird ages. PBS: birds were gavaged with sterile 1× phosphate-buffered saline. Cc and Cj preceding the strain number indicate *C. coli* and *C. jejuni*, respectively.

One room housed the control birds (gavaged with PBS) and three rooms housed the turkeys dual inoculated with *Campylobacter* spp.

*Campylobacter* inocula were prepared as follows: Each strain was cultured at 42°C in a 10% CO_2_ incubator on selective CLA-S medium for 48h and a loopful (approx. five colonies) was transferred into biphasic media in CELLSTAR^®^ 25-cm^2^ tissue culture flasks (Greiner Bio-One North America Inc., United States) including CLA-S and Bolton broth (5ml/5ml) and cultured without shaking under the same conditions. The next day, a loopful (10μl) of the inoculated Bolton broth was used to inoculate two CELLSTAR^®^ 75-cm^2^ tissue culture flasks (Greiner Bio-One North America Inc., United States) with the same biphasic media (10ml/10ml). Cultures were grown without shaking at 42°C microaerobically overnight. On the day of challenge, broth cultures were adjusted to an OD_600_ value of 0.4, centrifuged at maximum speed (5,251×g) for 10min, and resuspended in an identical volume of PBS. *Campylobacter* motility was assessed using dark-field microscopy as previously described (Sylte et al., 2019) and confirmed for all inocula.

### Enumeration of *Campylobacter* in Inoculum and Cecal Contents

Colony forming units (CFU) in suspensions utilized as inoculum were enumerated by plating appropriate dilutions on CAC and enumerating colonies following incubation at 42°C in a 10% CO_2_ incubator. To enumerate *Campylobacter* in cecal contents, 1g of cecal material was diluted in 9ml of sterile PBS (making the 10^−1^ dilution), vortexed for 5s, and serially diluted to 10^−6^. Using the track-plating dilution method (Jett et al., 1997; Siragusa, 1999), we plated 10μl of each dilution in duplicate on CAC to which appropriate antibiotics were added as needed (see Supplementary Table S1). The plated volume was increased from 10μl to 100μl for the recovery of NES on selective media (Day 15 and 22). Plates were incubated for up to 72h at 42°C in a 10% CO_2_ incubator and magenta colonies (*Campylobacter*) were enumerated to calculate CFU/g of cecal contents. For statistical purposes, if no putative *Campylobacter* colonies were obtained on selective media, the CFU was assigned the value of 10^3^CFU/g of cecal contents, i.e., the limit of detection (Sylte et al., 2018).

### DNA Extraction, Library Preparation, and Sequencing

From selective media showing NES, a single colony was picked and subcultured on biphasic media (MH) overnight at 42°C in a 10% CO_2_ incubator and used for DNA extraction using the PureLink^™^ Genomic DNA kit (Invitrogen, Carlsbad, CA) following the manufacturer’s instructions. DNA concentrations were determined using a NanoDrop^™^ spectrophotometer (Thermo Fisher Scientific) and the Qubit^®^ dsDNA Broad Range Assay with the Qubit Fluorimeter (Qiagen), and normalized to 200ng input DNA in 30μl final volume for Illumina MiSeq sequencing; 400ng DNA in 7.5μl final volume for sequencing using an Oxford Nanopore MinION standard flow cell; and 200ng DNA in 3.75μl final volume for sequencing using an Oxford Nanopore MinION Flongle flow cell.

DNA libraries were generated with the Nextera^™^ DNA Flex Library Prep kit (Illumina, San Diego, CA) according to the manufacturer’s instructions for MiSeq sequencing. For MinION sequencing, DNA libraries were generated with the rapid barcoding kit (SQK-RBK004; Oxford Nanopore, Oxford, United Kingdom) according to the manufacturer’s instructions.

Genome sequencing was performed at USDA, NADC, and Ames on a paired-end 300PE MiSeq (Illumina) instrument using MiSeq Reagent kit v3 and on a MinION instrument (Oxford Nanopore) using a standard flow cell (FLO-MIN106 R9.4.1) or a Flongle flow cell (FLO-FLG106). The MinION flow cell was run for 48–72h, and the resultant reads with a quality score > Q7 were either base called live in MinKNOW or afterward with Guppy v. 3.1.5 (Wick et al., 2019).

### Bioinformatic Analysis

#### Assembly

Multiple web-based bioinformatics tools available on the online Galaxy platform^1^ (Afgan et al., 2018) were used to generate genome assemblies. FastQC Galaxy Version 0.72+galaxy1 (Andrews, 2010) was used to inspect the quality of the Illumina reads. Short-read (MiSeq, Illumina Inc.) and long-read (MinION^™^ Nanopore Inc.) sequences were assembled using Unicycler Galaxy Version 0.4.8.0 (Wick et al., 2017). For one isolate, Unicycler was unable to produce a complete genome and we generated a complete consensus assembly using the Trycycler pipeline^2^ – from assemblies produced by flye (Kolmogorov et al., 2019), miniasm (Wick and Holt, 2019), and raven^3^ – which was then polished with pilon (Walker et al., 2014). Assembled genomes were annotated using Prokka Galaxy Version 1.14.5 (Seemann, 2014). Default parameters were used for all software, except for the parameter – min_fasta_length which was set to 1,000 in Unicycler.

#### Pangenome Analysis

To determine the parental strain that served as a recipient or a donor for a specific NES (i.e., carrying resistance markers of both parental strains), the average nucleotide identity (ANI) between each of the parental genomes and the relevant NES was calculated with pyani (Pritchard et al., 2016). The parental genome with the greatest ANI relative to the NES was identified as the recipient. Next, for each *in vitro* experiment, three genomes (two parental and one NES) were included in a pangenome analysis using Roary (Page et al., 2015). The stringency for assigning coding sequences to a gene was set to 99% identity. The tool gifrop^4^ (v0.0.6) was used to help identify transfer of genes or consecutive blocks of genes (hereafter referred to as a “genomic island”) absent in the recipient strain but present in the NES. The workflow used to complete this analysis is available online.^5^

#### Genotypic Analysis for AMR and Virulence

The resistance determinants of *Campylobacter* parental strains were determined using the web-based tool ResFinder 3.1^6^ (Zankari et al., 2012). Contigs (FASTA files) from each sample were screened for (i) chromosomal point mutations (both known and unknown) and (ii) acquired resistance genes with the default settings of 90% minimum identity and 20% minimum template length. For successful co-culture experiments, genomes were further screened for AMR and virulence genes using ABRicate version 1.0.1, which includes the databases ResFinder, CARD, ARG-ANNOT, and NCBI (Zankari et al., 2012; Gupta et al., 2014; Jia et al., 2017; Feldgarden et al., 2019). For ABRicate hits, the Prokka annotations were replaced with those from ABRicate. The NCBI AMRfinder tool (v3.9.8; Feldgarden et al., 2019) was also used to validate point mutation-based resistances.

## Results

Several types of selective media were tested to recover parental strains and NES. Parental strain Cc 6461, resistant to streptomycin but sensitive to ampicillin (see resistance patterns below), did not grow on CAC or MHA supplemented with streptomycin and ampicillin but grew on CLA-S with the same added antibiotics, i.e., showing antagonistic interactions between the antibiotics already present in CLA-S and the additional antibiotics. For this reason, CLA-S usage was discontinued during the course of the study and CAC was exclusively employed.

### Resistance Patterns of Wild-Type *Campylobacter* Strains

Phenotypic and genotypic resistance patterns of *Campylobacter* strains used in the co-culture experiments are summarized in Table 3, and minimum inhibitory concentration (MIC) results are provided in Supplementary Table S2. All strains exhibited resistance to one or more antimicrobials. Our genomic analyses detected streptomycin resistance either *via* the previously described *rpsL* K88R substitution (Olkkola et al., 2010) in Cj 6535 and Cj 6631, or *via aadE* (Pinto-Alphandary et al., 1990) in Cc 6461 (Table 3). Our analyses also identified two genes associated with aminoglycoside resistance in different strains: *aph(2″)-If* associated with both kanamycin and gentamicin resistance (Yao et al., 2017), and *aph(3′)-IIIa* associated with resistance to kanamycin but not to gentamicin (Gibreel et al., 2004; Crespo et al., 2016; Table 3). Erythromycin-resistant strain Cc 6461 showed the known substitution A2075G in all three copies of the 23S rRNA gene (Vacher et al., 2003); *erm(B)* associated with erythromycin resistance in certain *Campylobacter* strains (Qin et al., 2014) was not identified. The L22 A103V substitution associated with low-level macrolide resistance (Payot et al., 2006) was in the genome of two strains, Cc13150 and CjJCC, even though phenotypic macrolide resistance was not detected (erythromycin MICs 0.5μg/ml and 0.12μg/ml, respectively). Nalidixic acid (quinolone) resistance was associated with the *gyrA* T86I substitution (Wang et al., 1993) in the genome of three strains. Of note, this was identified using the AMRFinder tool (Feldgarden et al., 2019) but not ResFinder in the genome of two strains (Table 3). A closer analysis revealed that the T86I substitution was mediated by T86I ACA>ATT instead of the typically observed T86I ACA>ATA.

**Table 3:** Antimicrobial resistance patterns of *Campylobacter* strains (*n*=11).

Species and strain ^1^	Phenotypic resistance	Chromosomal point mutations		Acquired antimicrobial resistance gene	
		Mutation	Predicted resistance phenotype	Resistance gene	Predicted resistance phenotype
Cj 15152A	AKG			*aph(2′)-If*	KG (aminoglycosides)
				*blaOXA*	A (beta-lactams)
Cj 15152B	AKG			*aph(2″)-If*	KG (aminoglycosides)
				*blaOXA*	A (beta-lactams)
Cc 13150^a^	AKQ	L22 A103V^b^	Macrolides (low)	*aph(3′)-IIIa*	K (aminoglycosides)
		*gyrA* T86I^c^	Q (quinolones)	*blaOXA*	A (beta-lactams)
				*tet(O)* ^p^	T (tetracycline)
Cj 14398–5	AKG			*aph(2″)-If*	KG (aminoglycosides)
				*blaOXA*	A (beta-lactams)
Cj 11601MD	AKQT	*gyrA* T86I	Q (quinolones)	*aph(3′)-IIIa*	K (aminoglycosides)
				*blaOXA*	A (beta-lactams)
				*tet(O)* ^p^	T (tetracycline)
Cc 6461	TSE	23S A2075G	E (macrolides)	*aadE-Cc*	S (aminoglycosides)
				*tet(O)*	T (tetracycline)
Cj 14229–5	AKGT			*aph(2″)-If*	KG (aminoglycosides)
				*blaOXA*	A (beta-lactams)
				*tet(O)*	T (tetracycline)
Cj JCC	T	L22 A103V^b^	Macrolides (low)	*tet(O)* ^p^	T (tetracycline)
Cj 6535	ATS	*rpsL* K88R	S (aminoglycoside)	*blaOXA*	A (beta-lactams)
				*tet(O/32/O)* ^p^	T (tetracycline)
Cj 6631	ATS	*rpsL* K88R	S (aminoglycoside)	*blaOXA*	A (beta-lactams)
				*tet(O/32/O)* ^p^	T (tetracycline)
Cc 6067	AQT	*gyrA* T86I^c^	Q (quinolones)	*blaOXA*	A (beta-lactams)
				*tet(O)*	T (tetracycline)

1 Strains highlighted in gray have been sequenced previously (Dutta et al., 2016).

a Strain 13150 is phenotypically susceptible to tetracycline but carries a plasmid-borne *tet(O)*.

b L22 mutation was identified using the NCBI AMRfinder tool.

c *gyrA T86I* substitution was identified using the AMRFinder tool but not ResFinder.

p Plasmid-borne *tet(O)*.

Cc, *Campylobacter coli*; Cj, *Campylobacter jejuni*; A, ampicillin/carbenicillin; E, erythromycin; G, gentamicin; K, kanamycin; Q, nalidixic acid; S, streptomycin; and T, tetracycline. Phenotypic resistance patterns were identified by plating the different strains on Mueller-Hinton agar supplemented with antibiotics. Genotypic resistance patterns – chromosomal point mutations and acquired antimicrobial resistance genes – were identified by sequencing the genomes of each strain (Illumina MiSeq and MinION Nanopore) and analyzing the contigs (obtained with Unicycler) using the ResFinder 3.1 web-based tool (available at https://cge.cbs.dtu.dk/services/ResFinder/), ABRicate, and the NCBI AMRfinder tool. “Chromosomal point mutations” refer to known mutations conferring a phenotypic resistance described under “predicted resistance phenotype.” Unknown mutations identified with ResFinder (in genes where known mutations occur; no predicted phenotype) are summarized in Supplementary Table S3. For the three previously sequenced strains highlighted in gray (Dutta et al., 2016), the reported AMR patterns were confirmed and additional information was obtained about beta-lactam resistance.

We obtained certain unexpected findings in the determination of AMR determinants. Specifically, Cc 13150 was phenotypically susceptible to tetracycline but harbored *tet(O)*, typically associated with tetracycline resistance in *Campylobacter* spp. (Taylor et al., 1987; Zhao et al., 2016).

### Resistance Patterns of “Newly Emerged Strains” (NES) Recovered From *in vitro* Experiments

**Figure 1:**
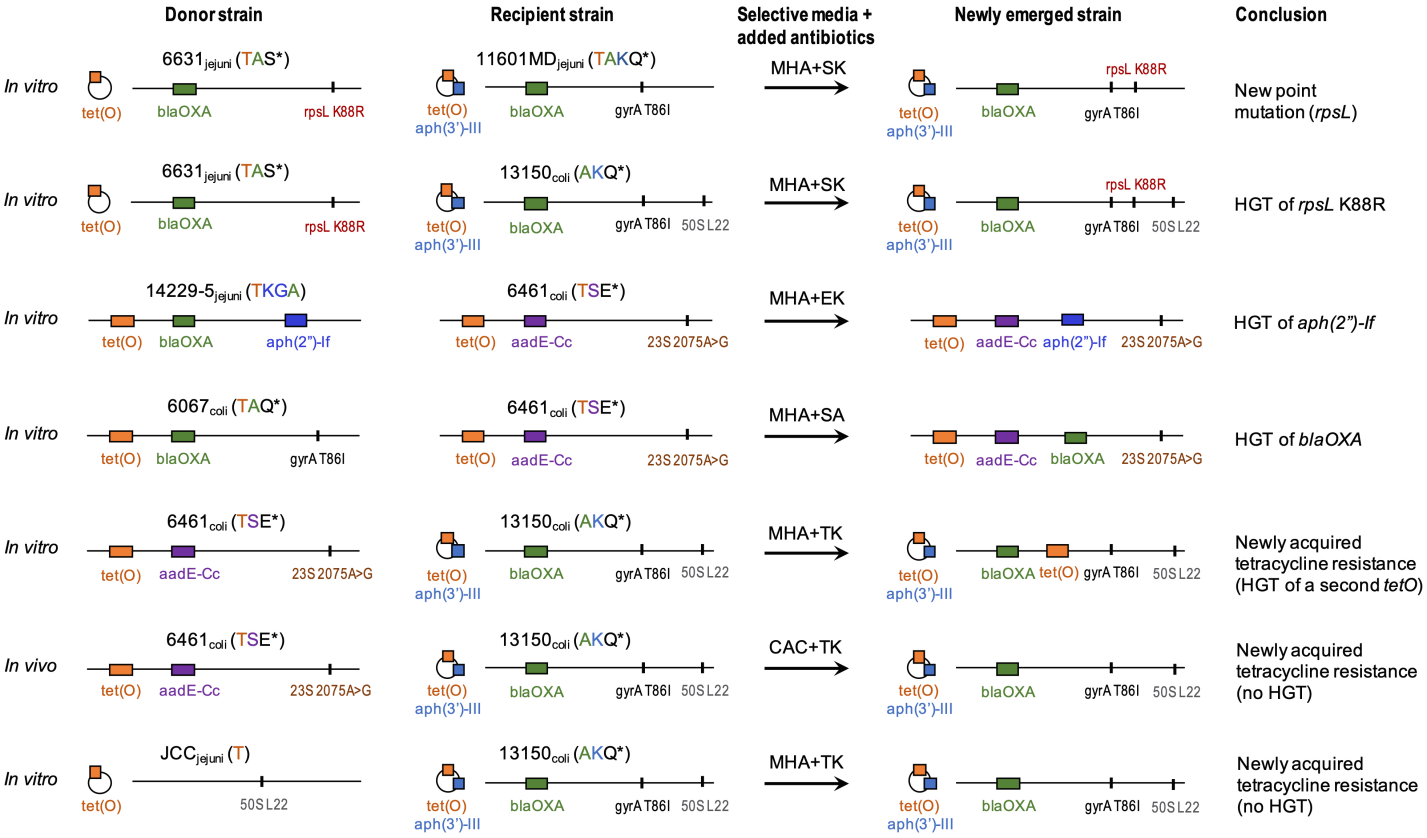
Summary of *in vitro* and *in vivo* experiments. Strains names are followed by *Campylobacter* species and phenotypic resistance. Acronyms include A, ampicillin/carbenicillin; E, erythromycin; G, gentamicin; K, kanamycin; Q, nalidixic acid; S, streptomycin; and T, tetracycline. ^*^indicates resistances due to a point mutation. Genotypic resistance can be carried on a chromosome (horizontal line) or a plasmid (circle). Resistance genes (colored blocks) are as: *tet(O)* (T resistance); *aph(3′)-IIIa* (K resistance); *blaOXA* (A resistance); *aadE-Cc* (S resistance); and *aph(2′)-If* (KG resistance). Point mutations (vertical lines) are as: *gyrA* T86I (nalidixic acid/ciprofloxacin: Q resistance); *23S* A2075G (E resistance); *rpsL* K88R (S resistance); and *L22* A103V (low macrolide resistance). Whole genome sequence data from newly emerged strain and parental strains were compared to identify donor *vs* recipient. CAC, CHROMagar^™^ *Campylobacter*; MHA, Mueller-Hinton agar; and HGT, horizontal gene transfer. Strain 13150 (phenotypically susceptible to tetracycline) carries a plasmid-borne non-functional *tet(O)* represented as a striped orange block.

Results from the 12 co-culture experiments are summarized in Table 4. In all co-culture experiments, we recovered both parental strains (data not shown). In six experiments, we also recovered isolates with a dual-resistance pattern, i.e., resistance markers of both parental strains (Figure 1). Putative NES had their genomes sequenced to characterize the resistance determinants.

**Table 4:** *Campylobacter* co-culture *in vitro*.

Parental strains (resistance pattern)		Antibiotics added to CAC	Putative NES	Recipient strain (NES pattern)	Acquired AMR
Cj 6631 (TS)	Cj 15152A (AKG)	TK	no		
Cj 6631 (TS)	Cj 15152B (AKG)	TK	no		
Cj 6631 (ATS^*^)	Cj 11601MD (AKTQ^*^)	SK	yes	Cj 11601MD (AKTS^*^Q^*^)	Point mutation *rpsL* (S resistance)
Cj 6631 (ATS^*^)	Cc 13150 (AKQ^*^)+*tet(O)*	SK	yes	Cc 13150 (AKS^*^Q^*^)+*tet(O)*	HGT of mutated *rpsL* (S resistance)
		SKQ	yes		
Cc 6461 (TE^*^S)	Cc 13150 (AKQ^*^)+*tet(O)*	TK	yes	Cc 13150 (A**T**KQ^*^)+*tet(O)*	Newly acquired phenotypic T / HGT *tet(O)*
		SA	no		
		SK	no		
		SKQ	no		
Cc 6067 (ATQ^*^)	Cc 6461 (TE^*^S)	SA	yes	Cc 6461 (**A**TE^*^S)	HGT *blaOXA* (A resistance)
Cj 6535 (ATS^*^)	Cj 11601MD (AKTQ^*^)	SK	no		
		SKQ	no		
Cj JCC (T)	Cj 14398–5 (AKG)	TK	no		
Cj JCC (T)	Cc 13150 (AKQ^*^)+*tet(O)*	TK	yes	Cc 13150 (AK**T**Q^*^)+*tet(O)*	Newly acquired phenotypic T / no HGT
Cj 14229–5 (AKGT)	Cc 6461 (TE^*^S)	SG	yes	Cc 6461 (TE^*^S**KG**)	HGT *aph(2″)-If* (KG resistance)
		EK	yes		
		SEK	yes		
		SKG	yes		
Cc 6461 (TE^*^S)	Cj 14398–5 (AKG)	SKG	no		
Cc 6461 (TE^*^S)	Cj 11601MD (AKTQ^*^)	SK	no		
		SKQ	no		

* indicates resistance due to a point mutation. Newly acquired resistance in NES compared to the parental recipient is in bold.

Cc, *Campylobacter coli*; Cj, *Campylobacter jejuni*; A, ampicillin/carbenicillin; E, erythromycin; G, gentamicin; K, kanamycin; Q, nalidixic acid; S, streptomycin; and T, tetracycline. The two parental strains were (i) co-cultured on Mueller-Hinton Agar (MHA), (ii) co-cultured on biphasic medium (5ml MHA+5ml Mueller-Hinton broth), or (iii) treated so that genomic DNA of one parental strain was mixed into colonies of the second parental strain on MHA. Resulting cultures were plated on selective media to recover newly emerged strains (NES). Whole genome sequence data from NES and parental strains were compared to identify donor vs recipient. The phenotypic resistance of each strain is provided in parentheses; Cc 13,150 which harbors *tet(O)* but does not express resistance to tetracycline is not indicated as “T.”

### Assessment of *in vitro* HGT *via* Experimental Dual Inoculation in Turkeys

Based on the results from the *in vitro* experiments, three *Campylobacter* pairs were chosen for further work *in vivo*. Turkeys were inoculated with the following strain pairs: Cc 6461 and Cj 14229–5 (room 9); Cc 6461 and Cc 13150 (room 10); and Cc 6461 and Cc 6067 (room 11; Table 2). The poults were free of *Campylobacter* prior to oral gavage and the control room (gavaged with PBS) remained free of *Campylobacter* throughout the experiment.

**Figure 2:**
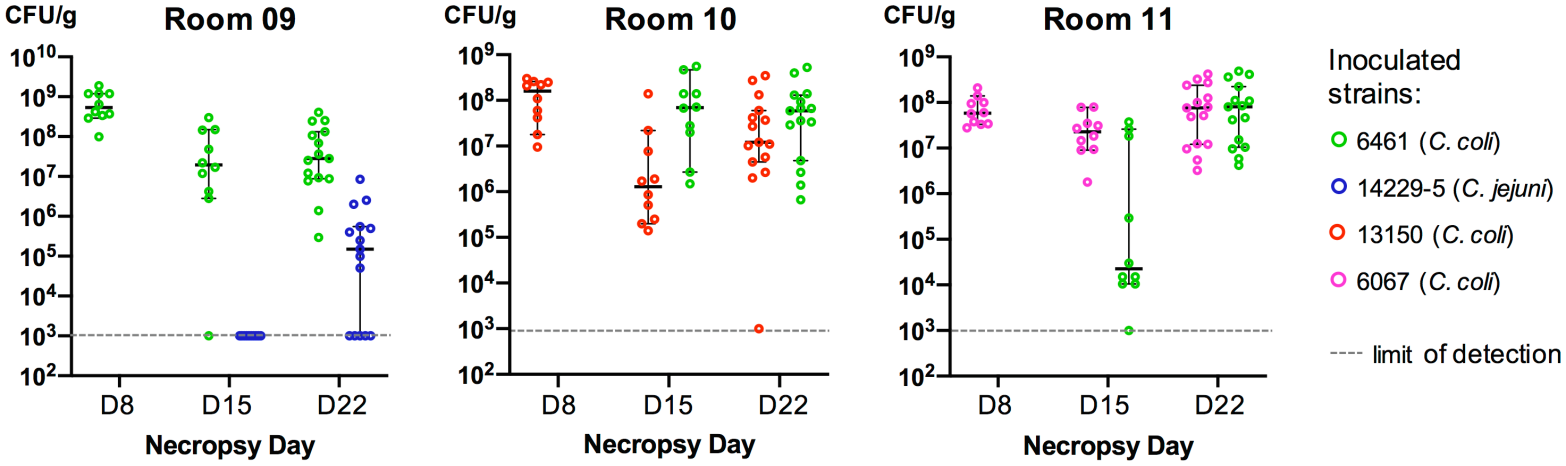
Cecal colonization levels (CFU/g cecal content) of *Campylobacter* in inoculated turkeys. Each bird was inoculated with two *Campylobacter* strains (one at Day 2 and the other at Day 9). Cc, *Campylobacter coli* and Cj, *Campylobacter jejuni*. In each room, 10–15 birds were euthanized at each necropsy and *Campylobacter* was recovered from cecal contents (see Methods). If no colonies resembling *Campylobacter* grew on selective media, the sample was assigned the value of 10^3^CFU/g of cecal contents (limit of detection). Mean CFU and 95% confidence intervals are represented (as bars) for each set.

On the first inoculation (Day 2), inoculum concentrations of the strains at the day of challenge were as: 3.8×10^10^CFU/ml (strain 6461, room 9); 4.7×10^9^CFU/ml (strain 13150, room 10); and 1.0×10^9^CFU/ml (strain 6067, room 11). In birds necropsied at Day 8, all three inoculated strains could be recovered from rooms 9 to 11 (Figure 2). On the second inoculation (Day 9), inoculum concentrations of the strains at the day of challenge were as: 10×10^8^CFU/ml (strain 14229–5, room 9); 5.5×10^8^CFU/ml (strain 6461, room 10); and 9.5×10^8^CFU/ml (strain 6461, room 11). In birds necropsied at Day 15, five of the six strains could be recovered from rooms 9 to 11, with strain 14229–5 not being recovered from room 9 on selective media (CAC with kanamycin; Figure 2). However, 1week later in birds necropsied at Day 22, both parental strains could be recovered from all necropsied birds (Figure 2).

On dual-selective CAC media designed to recover NES, we recovered a single colony from the cecal content of bird N3-R10-137 infected with Cc 6461 and Cc 13150 on CAC with tetracycline and kanamycin (TK). Cc 6461 was tetracycline resistant and carried a chromosomal *tet(O)*, while Cc 13150 was, as indicated above, phenotypically susceptible to tetracycline, despite carrying a plasmid-borne *tet(O)*. The hybrid resistance pattern (TK) in the NES was compatible with the acquisition of tetracycline resistance (T) from donor Cc 6461. The pangenome analysis indicated that the colonies recovered on TK were highly similar to the parental strain Cc 13150. A close comparison of the *tetO* regions in the parental strains and the NES suggested a homologous recombination event *via* which the non-functional *tet(O)* of Cc13150 was replaced by a functional *tet(O)* from donor strain Cc 6461 (Table 5; see details in “Pangenome analysis results”). The hypothesis that the defective *tet(O)* function was restored by a gene displacement is further supported by the detection of other homologous recombination events in other regions of the NES genome.

**Table 5:** Animal experiment results.

Parental strains (resistance pattern)		Antibiotics added to CAC	Putative NES	Recipient strain (NES pattern)	Acquired AMR
Cj 14229–5 (AKGT)	Cc 6461 (TE^*^S)	SG	no		
Cc 6461 (TE^*^S)	Cc 13150 (AKQ^*^)+*tet(O)*	TK	yes	Cc 13150 (A**T**KQ^*^)+*tet(O)*	Newly acquired phenotypic T/HGT *tet(O)*
Cc 6067 (ATQ^*^)	Cc 6461 (TE^*^S)	SA	no		

* indicates resistances due to a point mutation. New resistance in NES as compared to recipient is in bold.

Cc, *Campylobacter coli*; Cj, *Campylobacter jejuni*; A, ampicillin/carbenicillin; E, erythromycin; K, kanamycin; Q, nalidixic acid; S, streptomycin; T, tetracycline; and HGT, horizontal gene transfer. Phenotypic T: The NES is genetically similar to recipient strain 13150 (tetracycline susceptible) except it has a newly acquired tetracycline resistance. Turkeys were inoculated with two Campylobacter strains 7days apart. Cecal contents were plated after necropsy (Days 8, 15, and 22) on selective CHROMagar^™^ Campylobacter (CAC) plus additional antibiotics. The genome of recovered newly emerged strains (NES) – showing resistance markers of both parental strains – was sequenced and compared with both parental strains to identify donor vs recipient. The phenotypic resistance of each strain is provided in parentheses. Strain Cc 6461 carries *tet(O)* and is tetracycline resistant; strain Cc 13150 carries *tet(O)* but is tetracycline susceptible and thus not noted “T.”

### Pangenome Analysis Results

Overall, the emergence of a novel resistance trait in the recipient strains occurred *via* a spontaneous point mutation or *via* a recombination-mediated horizontal gene transfer. We observed different HGT events: (i) the transfer of a genomic island including an ARG that was previously absent or (ii) the allelic transfer of an ARG, i.e., the replacement of an ARG already present by a mutated version of the same gene *via* homologous recombination.

The pangenome analysis was used to determine which of the two strains in the co-culture were the source of the ARG *vs* the recipient of the acquired resistance and to highlight genomic differences between parental strains and the NES, i.e., gene or genomic island transfers (genes absent in the recipient but present in both donor and NES) and deletions (genes present in the recipient but not the NES).

#### Mutation-Mediated Novel Resistance

In the *in vitro* co-cultures of Cj 6631 and Cj 11601MD, the NES recovered on KS was highly similar to the parental strain Cj 11601MD with an additional mutated version of gene *rpsL* (K88R) conferring resistance to streptomycin (Figure 1). To differentiate between the horizontal transfer of K88R *rpsL* from Cj 6631and a novel spontaneous mutation in Cj 11601MD, we compared the *rpsL* sequences between the strains. Aside from the K88R mutation, Cj 6631 *rpsL* sequence included two single-nucleotide polymorphisms that were absent in the Cj 11601MD and the NES genomes. Because the rest of the NES genome was also highly similar to Cj 11601MD, this suggest a spontaneous point mutation in Cj 11601MD, rather than the displacement of a mutated *rpsL* from Cj 6631.

**Figure 3:**
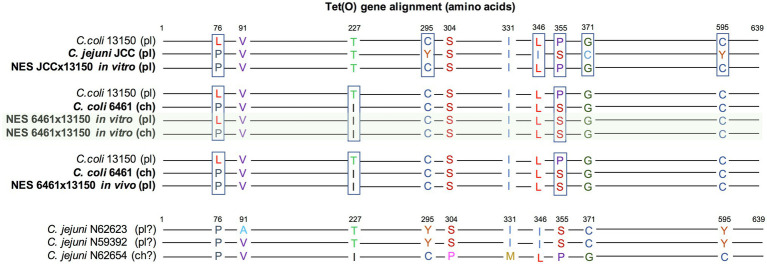
Alignment of the *tet(O)* genes of *Campylobacter* strains. Parental strains Cc 6461, Cc 13150, and Cj JCC were compared to newly emerged strains (NES) recovered from *in vitro* co-culture experiments and the experimental infections of turkeys. Three previously published tetracycline-susceptible strains carrying *tet(O)* (Whitehouse et al., 2018) were included for comparison (BioProject PRJNA292664; accession numbers N59392, N62623, and N62654). The *tet(O)* gene was either plasmid-borne (pl) or chromosomal (ch; there is some uncertainty noted “?” for the public strains as the draft genomes are broken into many contigs). The tetracycline-resistant NES recovered from the *in vitro* co-culture of Cc 6461 and Cc 13150 carried two copies of the *tet(O)* gene, one chromosomal [corresponding to the chromosomal *tet(O)* of Cc 6461 (Crespo et al., 2012)] and one plasmid-borne (highlighted in green). The “nonfunctional” version of Cc 13150 has a leucine at residue 76, whereas the “functional” versions have a proline at residue 76.

In *in vitro* co-cultures of Cj JCC and Cc 13150, the NES recovered on TK (compatible with the acquisition of tetracycline resistance from donor Cj JCC) was highly similar to parental strain Cc 13150 but expressing resistance to tetracycline (Figure 1). Sequencing revealed that all three strains (parental and NES) carried *tet(O)* commonly associated with tetracycline resistance in *Campylobacter* (Sougakoff et al., 1987), despite Cc 13150 being phenotypically susceptible to tetracycline (MIC=0.25μg/ml). We compared the “functional” and the “nonfunctional” versions of *tet(O)* and found that the “nonfunctional” version (in Cc 13150) had a leucine at residue 76, whereas the “functional” versions had a proline at this position (Figure 3). The observed *tet(O)* L76P substitution seems to restore the functionality of *tet(O)* (i.e., tetracycline resistance) in the NES. Of note, when adding three published tetracycline-susceptible strains carrying a “nonfunctional” *tet(O)* to the comparison (Whitehouse et al., 2018), a proline was identified at residue 76 of the *tet(O)* gene, and *tet(O)* in one of these strains (N59392) was identical to *tet(O)* of the tetracycline-resistant strain Cj JCC (Figure 3); hence, an explanation other than the L76P mutation must be found for these three published tetracycline-susceptible strains that harbor *tet(O)*.

#### Horizontal Transfer of Resistance Genes

In co-cultures of Cc 6461 and Cc 13150, the pangenome analysis detected the transfer of a chromosomal genomic island including *tet(O)* (2661bp) from donor Cc 6461 to the chromosome of the recipient Cc 13150 (chromosomal), leading to two copies of *tet(O)* in the NES, one chromosomal, newly acquired, and the original plasmid-borne copy (Figures 1, 3; Table 6). Of note, the plasmid-borne *tet(O)* in the NES differed from the plasmid-borne *tet(O)* in strain Cc 13150 by two amino acids. It was not possible to determine if this was due to a double mutation in the original gene or a displacement with a *tet(O)* from donor Cc 6461 with an additional point mutation (Figure 3).

**Table 6:** Detailed information regarding genomic islands transfers.

Experiment	Start	End	Island length	Number genes	Genes transferred	Annotation
6631×13150 *in vitro*	433,527	439,905	6,378	8	***rpsL** rpsG fusA* group_1637 group_1638 group_1639 group_1640 *trpC*	**30S ribosomal protein S12** (*K88R-mutated*) 30S ribosomal protein S7 elongation factor G exporting protein methyltransferase domain-containing protein hypothetical protein lipoprotein indole-3-glycerol phosphate synthase
14229–5×6461 *in vitro*	488,562	493,264	4,703	6	group_363 **group_364** group_368 group_367 group_366 group_365	IS200/IS605 family transposase IS605 **aminoglycoside O-phosphotransferase APH(2″)-If** hypothetical protein IS1595 family transposase ISAcsp6 hypothetical protein hypothetical protein
6067×6461 *in vitro*	1,502,456	1,504,198	1,743	2	**group_177** *btuD*	**beta-lactamase OXA-133** vitamin B12 import ATP-binding protein BtuD
6461×13150 *in vitro*	140,166	142,826	2,661	2	***tet(O)*** group_901	**tetracycline resistance ribosomal protection protein Tet(O)** hypothetical protein

For each experiment, three genomes (from the two parental strains and one “newly emerged strain”) were included in a pangenome analysis using Roary (Page et al., 2015). Antimicrobial resistance genes and resistance-conferring mutated genes are in bold. Of note, genes present in both parental strains and the newly emerged strain, and contiguous to the transferred genes might have also been transferred as part of the AMR genomic island – but undetectable by the analysis because they are 100% identical, suggesting that the “island length” below is underestimated.

A different outcome was observed in the *in vivo* dual inoculation of the same two strains, Cc 6461 and Cc 13150. Instead of the acquisition by Cc 13150 of a second copy of *tet(O)* detected *in vitro*, we observed *in vivo* the displacement of the non-functional *tet(O)* present in the plasmid of recipient strain Cc 13150 by the functional *tet(O)* from donor strain Cc 6461 by homologous recombination.

Additional instances of HGT of resistance genes were noted. In co-cultures of Cc 6461 and Cc 6067, the NES recovered on SA (compatible with either the acquisition of streptomycin resistance from Cc 6461 or the acquisition of ampicillin resistance from Cc 6067) showed the acquisition of ampicillin resistance *via* transfer of a genomic island including *blaOXA* (1743bp) from donor Cc 6067 to recipient Cc 6461 (Figure 1; Table 6). In co-cultures of Cc 6461 and Cj 14229–5, the NES recovered on EK showed the acquisition of kanamycin/gentamicin resistance *via* the transfer of a genomic island (4703bp) including *aph(2″)-If* from donor 14229–5 to recipient 6461 (Figure 1; Table 6).

In addition to acquisition of new ARGs, we noted HGT-mediated transfer of genes harboring specific substitutions associated with resistance. For instance, in co-cultures of Cj 6631 and Cc 13150, the NES recovered on KS was highly similar to the parental strain Cc 13150 with an additional K88R-mutated version of *rpsL* (Figure 1). The pangenome analysis confirmed the acquisition of streptomycin resistance *via* the transfer of a genomic island (6378bp) including a K88R-mutated version of *rpsL* from donor Cj 6631 to recipient Cc 13150, and not a novel spontaneous mutation of *rpsL* (Table 6).

In the co-culture of Cc 6461 and Cc 6067, several virulence-associated genes (*cheW*, *cheA*, *cheV*, *fliF*, and *fliG*) were also identified in close proximity but not contiguous to the transferred genomic island including the resistance gene *blaOXA*, while in the co-culture of Cc 6461 and Cc 13150, no virulence genes were identified in close proximity to the genomic island that harbored the resistance gene *tet(O)* (Supplementary Figure 1).

Of note, aside from the resistance genes transfers, each NES showed various other transfer or deletion events that are not fully detailed here (see Supplementary Table S4 for details).

### Reproducibility of the Horizontal Transfer of Resistance Genes

**Figure 4:**
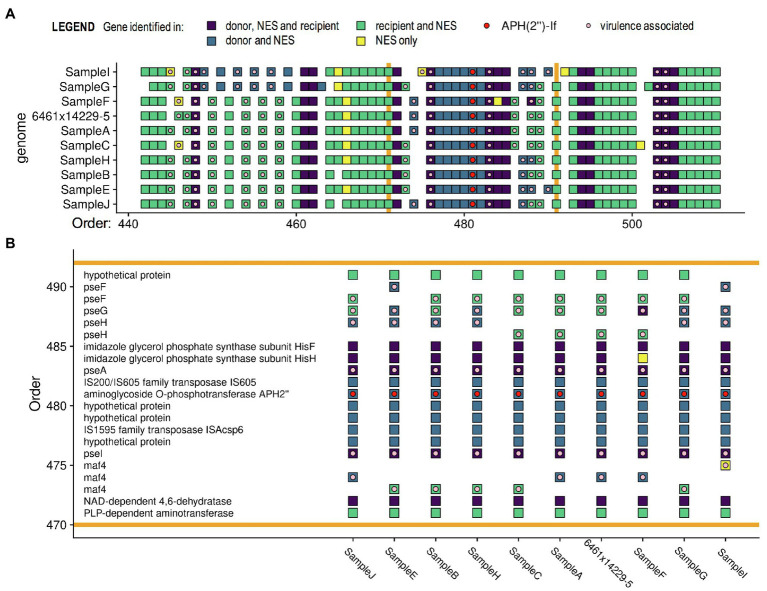
Pangenome comparisons of 10 isolates of *in vitro* co-cultures of *C. coli* 6461 and *C. jejuni* 14229–5. **(A)** Only a section of the genomes is presented, highlighting **(B)** the transferred genomic island (between the orange bars) including resistance gene *aph(2′)-If* associated with kanamycin/gentamicin resistance. Each square represents a gene (annotated using PROKKA). The pangenome analysis was done using Roary, the virulence (light pink dot) and resistance genes (red dot) were annotated using ABRicate, and the figure was made with ggplot2 in “R.” Genes annotated as virulence genes are associated with *Campylobacter* motility. Of note, genes classified as the same gene in two strains have sequences ≥ 99% identical. Genes in yellow, i.e., found only in the newly emerged strain (NES), are genes that are less than 99% identical to the two parental genes, which might be due to chimeras of donor and recipient versions of the gene.

To determine reproducibility of the observed HGT events, we replicated the co-culture of Cc 6461 and Cj 14229–5, and subcultured and sequenced 10colonies (A–J) showing a dual-resistance pattern (SG). The NES originally sequenced and the nine newly sequenced NES from the replicate experiment (A–C and E–J) showed the same transfer of a genomic island including the resistance gene *aph(2″)-If* and five other genes at the same location in the recipient genome, but some variability in other transfer and deletion events elsewhere in the genome (Figure 4 and Supplementary Table S4). We detected the presence of genes associated with *Campylobacter* motility (such as *pseA* and *pseI*) in close proximity to *aph(2″)-If* in all sequences, with additional virulence-associated genes (e.g., *maf4*) being transferred in six out of 10 isolates (Figure 4). Of note, genes present in both parental strains and the NES (in dark purple in Figure 4) and contiguous to the genomic island carrying the ARG (in blue in Figure 4) might have been transferred concomitantly with it but were invisible to the analysis because these genes were 100% identical in the parental strains and the NES. Such findings suggest that contiguous *pseA*, *pseI*, and *maf4* (involved in *Campylobacter* motility) were likely transferred with AMR as part of a single HGT, rather than multiple HGTs (Figure 4).

## Discussion

Despite the increasing prevalence of AMR in *C. jejuni* and *C. coli* in food animals, such as swine and poultry, as well as the potential impact to human campylobacteriosis treatment, there is limited information on *Campylobacter* AMR acquisition in animals co-infected with different *Campylobacter* strains. This is especially true for turkeys, which are known for carrying multidrug-resistant *Campylobacter* strains of both *C. coli* and *C. jejuni* (Wright et al., 2008; Wesley et al., 2009). Here, we used turkey dual inoculations to investigate AMR transfer between different *Campylobacter* strains and species (originating from swine and turkeys), after also investigating the process in simpler models involving co-cultures *in vitro*.

Using *in vitro* co-cultures, we identified the horizontal transfer of chromosomal genes conferring resistance to beta-lactam (*blaOXA*), aminoglycoside [*aph(2″)-If*; *rpsL* K88R], and tetracycline [*tet(O)*], as well as a spontaneous mutation (in *rpsL*) conferring a novel resistance to streptomycin. Our findings suggest that even though barriers exist to keep exogenous DNA (e.g., from gut commensals) from being incorporated in *Campylobacter* species – through CRISPR-Cas and restriction-modification systems (Gardner and Olson, 2012) – transformation can mediate the horizontal transfer of resistance determinants between *C. coli* strains, as well and between *C. jejuni* and *C. coli*, including between strains originating from different animal hosts (swine and turkey).

Both *C. coli* and *C. jejuni* are naturally competent, i.e., can take up naked DNA without any special treatment (Wang and Taylor, 1990). Interestingly, the HGT-mediated acquisition of previously absent resistance genes always occurred through the transfer of consecutive blocks of genes – genomic islands – *via* homologous recombination (i.e., in regions of the genome showing high similarity between the recipient and donor strains). Our study also found two occurrences of resistance-associated genes being displaced by a mutated version of the same gene through homologous recombination (HGT of a single gene). Interestingly, even though we detected *tet(O)*-carrying plasmids in several parental strains (Figure 1), we did not observe any transfer of such plasmids. This supports previous reports that transformation of *Campylobacter* with plasmid DNA is much less efficient than with chromosomal DNA (Wang and Taylor, 1990; Taylor, 1992). *In vitro* studies on the natural transformation of *Streptococcus pneumoniae* (Prudhomme et al., 2002) and *Acinetobacter* spp. (De Vries and Wackernagel, 2002) identified short (3–10 nucleotides) stretches of sequence identity (microhomology) between donor and recipient DNA serving as recombinational anchors, leading to homology-facilitated illegitimate recombination. For *S. pneumoniae*, transformation occurred preferentially within segments of high GC content (Prudhomme et al., 2002). Further experimental studies will be needed to identify the mechanisms involved in the homologous recombination of *Campylobacter* spp.

In our experiments, streptomycin resistance was acquired once through a K88R mutation of *rpsL*, and once through a horizontal transfer of a K88R-mutated *rpsL*. The horizontal transfer of mutated genes was expected as previous studies have suggested that, in the absence of selective pressure, the frequency of transformation to resistance greatly exceeds the frequency of spontaneous mutations to resistance (transformation to resistance was observed with mutated *rpsL* (Oyarzabal et al., 2007), 23S rRNA (Kim et al., 2006), and *gyrA* (Wilson et al., 2003; Kim et al., 2008).

Of note, the acquisition of a genomic island including a K88R-mutated version of *rpsL* in the NES recovered from the co-culture of Cj 6631 and Cc 13150 was only detected after manually checking the *rpsL* region in order to confirm the occurrence of a spontaneous point mutation K88R. From the pangenome perspective, *rpsL* was more than 99% identical in all genomes (one nucleotide difference between the mutated and non-mutated gene) and hence was excluded from the list of possibly transferred genes. This also happened when trying to detect a mutated version of *tet(O)* (which restored the gene functionality) in the NES recovered *in vivo* (6461×13150). The pangenome framework can struggle to identify allelic transfers, such as these, where allelic versions of the gene conferring resistance are highly similar.

*In vitro* results were highly reproducible – co-culture of specific pairs consistently yielded NES recovered on selective media while other pairs consistently yielded no NES recovered – suggesting that HGT events are not random and/or that some strains might be more prone to horizontal gene transfers. In NES sequenced from co-cultures of Cc 6461 and Cj 14229–5, the horizontal transfer of a resistance gene consistently occurred through the transfer of the same genomic island, and at the same place on the recipient genome, showing that transformation is not random, and possibly indicating “recombination hotspots” (Yahara et al., 2014).

Of note, Cc 6461, the only swine-derived strain in our panel, was involved in four HGT events (including in a turkey host) as a donor and as a recipient, which suggests this strain may be more prone to genetic exchanges with other strains. The ability of turkeys to be colonized by both *C. coli* and *C. jejuni* has triggered interest as well as concern because swine and turkey farms can be located in close proximity, which is the case in the southeastern United States where our *Campylobacter* strains were isolated (Wright et al., 2008; Bolinger et al., 2018). In our dual-inoculation experiment, co-colonization with different strains of *C. coli* (assessed by plating cecal content) was successful with no apparent dominance between the two strains; co-colonization with *C. jejuni* and *C. coli* also occurred even though the *C. jejuni* strain (inoculated second) was not recovered the first week after inoculation but was recovered 2weeks after inoculation. Turkeys, if co-colonized by *C. coli* and *C. jejuni*, could be a host species where resistance transfers between the two species are more likely to occur and be further transferred to other animal host species. The possible transfer of resistance between bacteria in swine and turkeys warrants further investigation.

Interestingly, in two out of five experiments that showed the acquisition of resistance through HGT, the transfer occurred in close proximity to genes related to chemotaxis or the biosynthesis of functional flagella, and thereby to motility and pathogenesis of *Campylobacter* (Guerry et al., 2006; Salah Ud-Din and Roujeinikova, 2018). Genes associated with motility/colonization are frequently recombining genes and are considered to contribute to the pathogen’s adaptive potential (Park et al., 2020). Hence, the co-localization of ARGs near such motility genes on recombination hotspots could impact the transmission rate of ARGs through the population.

Sequencing strain Cc 13150, phenotypically susceptible to tetracycline, revealed a plasmid-borne *tet(O)*. Such a discrepancy between phenotypic and genotypic tetracycline resistance in *Campylobacter* is uncommon but has been reported before, being noted in four out of 327 *tet(O)*-harboring isolates (Whitehouse et al., 2018). In three experiments involving strain Cc 13150, a newly acquired phenotypic resistance to tetracycline was observed in NES. Different mechanisms were involved as: a *tet(O)* P76L mutation that restored the function of the gene present in a plasmid in Cc 13150, or the acquisition of a new (functional) *tet(O)* gene by HGT. In one HGT event, a second (chromosomal) *tet(O)* was acquired, while in the other HGT, the non-functional *tet(O)* present in Cc 13150 was replaced by a functional *tet(O)* originating from donor Cc 6461 by homologous recombination.

Our study provides evidence for the natural horizontal transfer of a chromosomal *tet(O)* between different *C. coli* strains in the turkey intestine. During the *in vivo* experiment, birds were not inoculated with both strains at the same time to ensure that any resistance gene transfer would occur in the animal intestine (post-colonization) rather than during a co-inoculation. From the cecal contents of dual-strain inoculated birds, both parental strains were recovered, as well as a single NES from a bird that was inoculated with strains Cc 6461 and Cc 13150. In the recovered NES – with a newly acquired tetracycline resistance gene compared to the recipient strain – we detected a horizontal gene transfer on a plasmid, leading to the displacement of a non-functional *tet(O)* by a functional *tet(O)* by homologous recombination. This result was slightly different from the *in vitro* co-culture result where a second (chromosomal) *tet(O)* was acquired.

Several hypotheses could explain why other horizontal transfers of ARGs observed *in vitro* were not detected *in vivo*. Firstly, transformation between different *Campylobacter* strains and species is likely a rare event and it might be more difficult to recover recombinant strains from complex environments, such as cecal contents. Secondly, laboratory conditions are markedly different from the environment encountered by *Campylobacter* colonizing a turkey cecum. *In vivo*, naked DNA may become degraded by extracellular nucleases of other cecal microbes, reducing the frequency of transformation. Finally, transmission and adaptation of a new phenotype may take longer than our study – turkeys are reared up to 21weeks of age before slaughter – or transformation frequency might be lower *in vivo*.

This work provides further evidence that transformation can mediate the *in vitro* and *in vivo* HGT of antimicrobial resistance genes in *Campylobacter*, within and between species (*C. coli* and *C. jejuni*). *In vitro*, resistance gene transfer was not a rare event and was highly reproducible between specific strains, and homologous recombination seemed to be a mechanism of choice for resistance spread. Finally, some strains seem more prone to the acquisition or transfer of resistance genes. Being able to identify such strains could be important for prevention of antimicrobial resistance transmission.

## Data Availability Statement

The datasets presented in this study can be found in online repositories. The names of the repository/repositories and accession number(s) can be found in the article/Supplementary Material.

## Ethics Statement

The animal study was reviewed and approved by the National Animal Disease Center (NADC) Institutional Animal Care and Use Committee (IACUC).

## Author Contributions

Project design and coordination were performed by TL. ZH isolated the *Campylobacter* strains used in the study and tested them for phenotypic resistance. VG-C performed the *in vitro* experiments, DNA extraction, sample library preparation, and sequencing. TL, VG-C, JM, JQ, and MS performed the animal experiment. JT and VG-C performed the bioinformatics analyses. VG-C wrote the initial manuscript and later versions were based on input and suggestions from all. All authors contributed to the article and approved the submitted version.

## Funding

This research was supported by the United States Department of Agriculture-Agricultural Research Service (USDA-ARS) funds under project 5030-31320-004-00D. Partial support was also provided by the USDA-NIFA award 2018-67017-27927. Mention of trade names or commercial products in this publication is solely for the purpose of providing specific information and does not imply recommendation or endorsement by the USDA. USDA is an equal opportunity provider and employer. The Galaxy server that was used for some bioinformatics calculations is in part funded by the Collaborative Research Centre 992 Medical Epigenetics (DFG grant SFB 992/1 2012) and the German Federal Ministry of Education and Research [BMBF grants 031 A538A/A538C RBC, 031L0101B/031L0101C de.NBI-epi, and 031 L0106 de.STAIR (de.NBI)].

## Conflict of Interest

The authors declare that the research was conducted in the absence of any commercial or financial relationships that could be construed as a potential conflict of interest.

## Publisher’s Note

All claims expressed in this article are solely those of the authors and do not necessarily represent those of their affiliated organizations, or those of the publisher, the editors and the reviewers. Any product that may be evaluated in this article, or claim that may be made by its manufacturer, is not guaranteed or endorsed by the publisher.

## Footnotes

^1^ https://usegalaxy.org/

^2^ https://github.com/rrwick/Trycycler

^3^ https://github.com/lbcb-sci/raven

^4^ https://github.com/Jtrachsel/gifrop

^5^ https://github.com/Jtrachsel/Campy_HGT

^6^ https://cge.cbs.dtu.dk/services/ResFinder/

## Supplementary Material

**SUPPLEMENTARY FIGURE S1** | Results of the pangenome analysis of *in vitro* co-culture of Campylobacter strains. Only a section of the genome is presented, highlighting the region surrounding the resistance gene of interest. (S1A) Co-culture of 6461 and 6067; (S1B and S1C) Co-culture of 6461 and 13150. Of note, the newly emerged strain carries two copies of *tet(O)*, one chromosomal (S1B) and one plasmidic (S1C).

**SUPPLEMENTARY TABLE S1** | List of media with combination of antibiotics used for the plating of turkey cecal content by room and necropsy day. Different combinations were used to recover one (at Day 8) or two parental strains (Day 15 and Day22) as well as a possible newly emerged strain with dual resistance.

**SUPPLEMENTARY TABLE S2** | Minimal inhibitory concentrations (MICs) from CAMPY2 Sensititre^®^ plates (VersaTREK Diagnostics/Thermo Fisher, United States). Antimicrobial levels are given in μg/ml. Eight antimicrobials were tested. S, susceptible; R, resistant; and NI, no interpretation because guidelines have not been established by the Clinical and Laboratory Standards Institute (CLSI) for the bacteria/drug combination being tested. Testing was conducted by the Veterinary Diagnostic Laboratory at ISUVDL. Sensitivity testing failed for strain 14229–5, the result provided being “Isolate was unable to grow for sensitivity (two attempts).”

**SUPPLEMENTARY TABLE S3** | Unknown mutations (in genes where specific known mutations confer resistance) in *Campylobacter* sp. as identified using the web-based tool ResFinder 4.1^7^ (Zankari et al., 2012). Contigs (FASTA files) from each sample were screened for “Chromosomal point mutations - Unknown mutations” with the default settings of 90% minimum identity and 20% minimum template length.

**SUPPLEMENTARY TABLE S4** | Detailed horizontal gene transfers observed in the newly emerged strains (NES). For each *in vitro* transfer experiment, three genomes (from two parental and one NES strain) were included in the pangenome analysis using Roary (Page et al., 2015). The stringency for assigning coding sequences to a gene was set to 99% identity. The tool gifrop^8^ (v0.0.6) was used to help identify transfer of genes or consecutive blocks of genes – hereafter referred to as a “genomic island” – absent in the recipient strain but present in the NES. The workflow used to complete this analysis is available online.^9^
