# Supplementary material for: Natural Horizontal Gene Transfer of Antimicrobial Resistance Genes in Campylobacter spp. From Turkeys and Swine
Source: Front Microbiol. 2021 Sep 27;12:732969. doi: 10.3389/fmicb.2021.732969 (PMC8504540; doi:10.3389/fmicb.2021.732969)

Supplementary Figure 1A.

Pangenome analysis of *in vitro* co-culture of 6461 and 6067. (A) Only a section of the genome is presented, highlighting (B) the transferred genomic island (between the orange bars) including resistance gene *blaOXA* associated with beta-lactam resistance. Each square represents a gene (annotated using PROKKA).The pangenome analysis was done using Roary, the virulence (light pink dot) and resistance genes (red dot) were annotated using ABRicate and the figure was made with ggplot2 in ‘R’. Genes annotated as virulence genes are associated with *Campylobacter* motility.

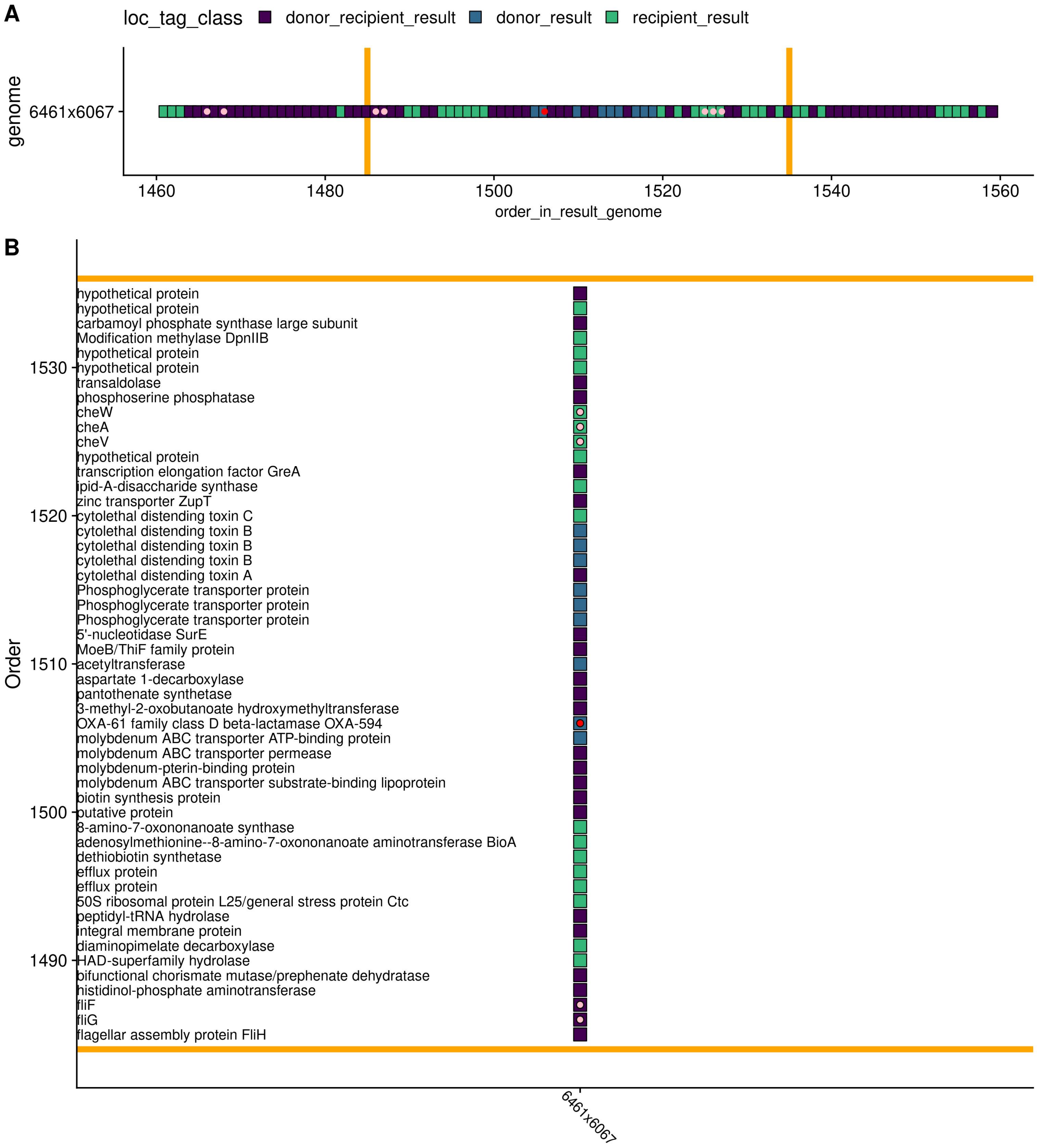

Supplementary Figure 1B.

Pangenome analysis of *in vitro* co-culture of 6461 and 13150. Of note, the newly emerged strain carries two copies of the tet(O) gene, one plasmidic and one chromosomal. This is the <u>chromosomal</u> one. (A) Only a section of the genome is presented, highlighting (B) the transferred genomic island (between the orange bars) including resistance gene *tet(O)* associated with tetracycline resistance. Each square represents a gene (annotated using PROKKA).The pangenome analysis was done using Roary, the virulence (light pink dot) and resistance genes (red dot) were annotated using ABRicate and the figure was made with ggplot2 in 'R'. Genes annotated as virulence genes are associated with *Campylobacter* motility.

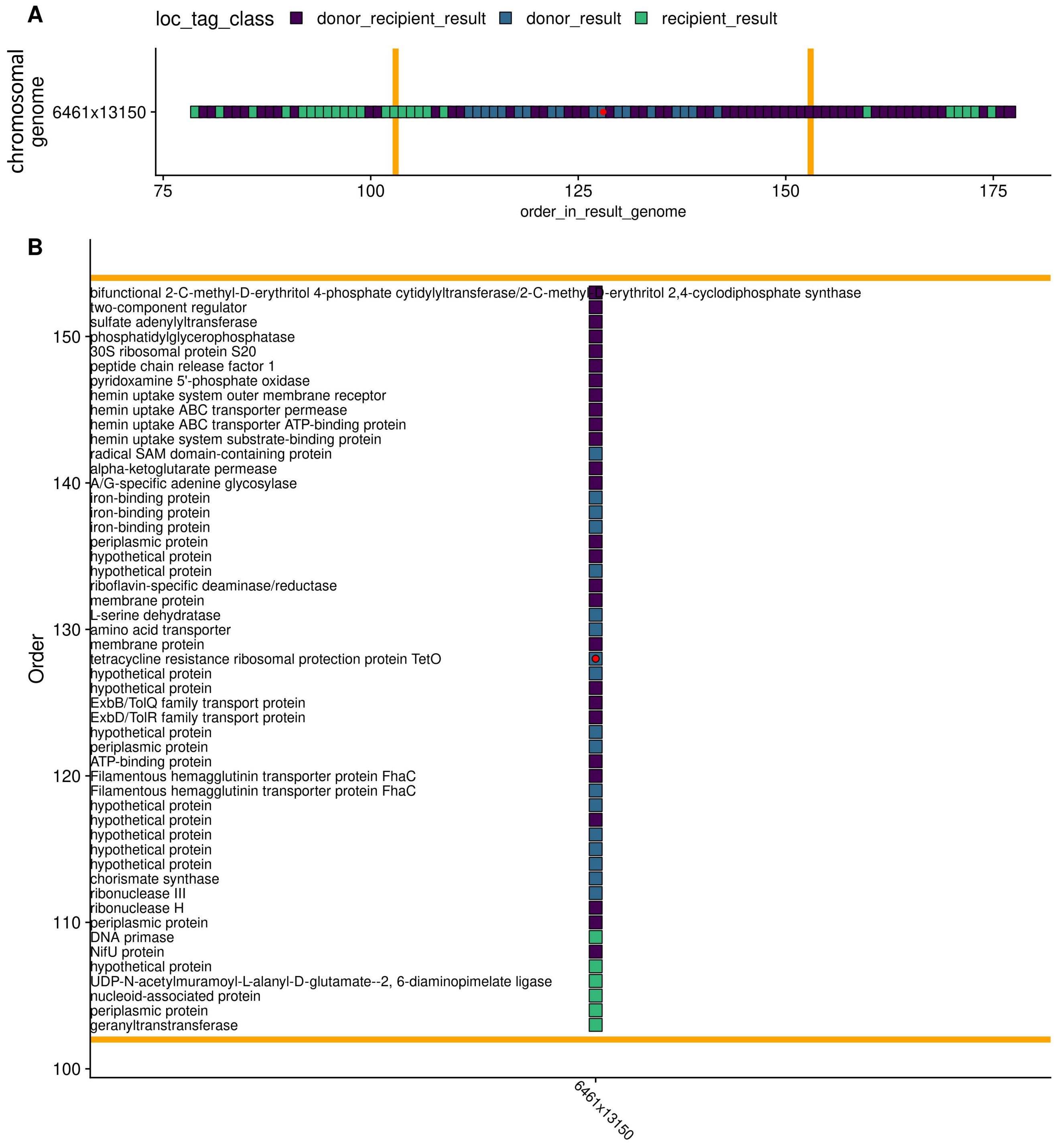

Supplementary Figure 1C.

Pangenome analysis of *in vitro* co-culture of 6461 and 13150. Of note, the newly emerged strain carries two copies of the tet(O) gene, one plasmidic and one chromosomal. This is the plasmidic one. Only a section of the genome is presented, highlighting the resistance gene *tet(O)* associated with tetracycline resistance and its surrounding genes. Each square represents a gene (annotated using PROKKA).The pangenome analysis was done using Roary, the virulence (light pink dot) and resistance genes (red dot) were annotated using ABRicate and the figure was made with ggplot2 in 'R'. Genes annotated as virulence genes are associated with *Campylobacter* motility.

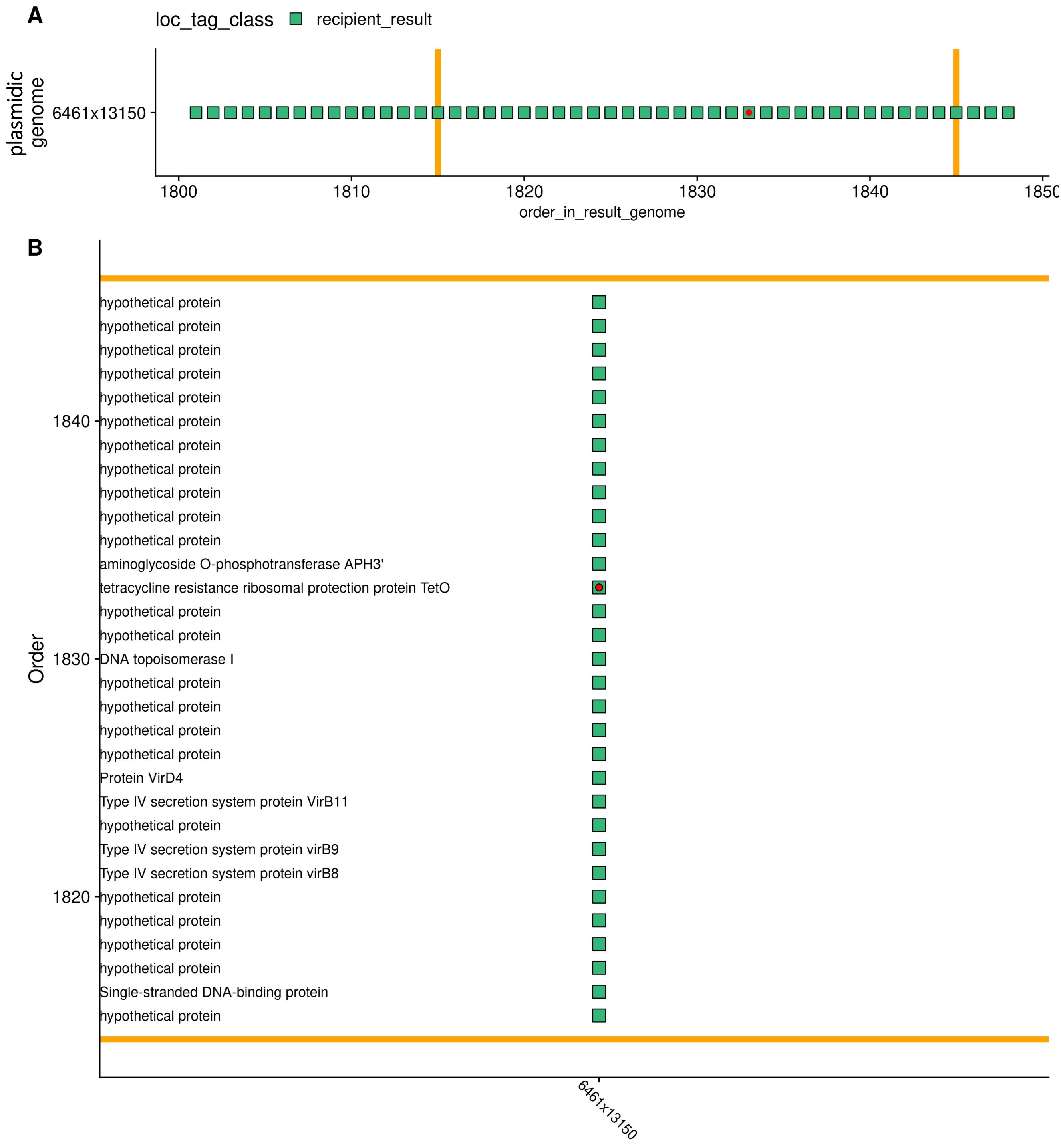

Supplementary Figure 1D.

Pangenome analysis of *in vivo* dual infection of 6461 and 13150 in turkeys. Only a section of the genome is presented, highlighting the resistance gene *tet(O)* associated with tetracycline resistance and its surrounding genes. Each square represents a gene (annotated using PROKKA).The pangenome analysis was done using Roary, the resistance gene (red dot) was annotated using ABRicate and the figure was made with ggplot2 in 'R'. The *tet(O)* gene was transferred by itself (not as part of a genomic island), and replaced the already present *tet(O)* gene (non-functional) through homologous recombination

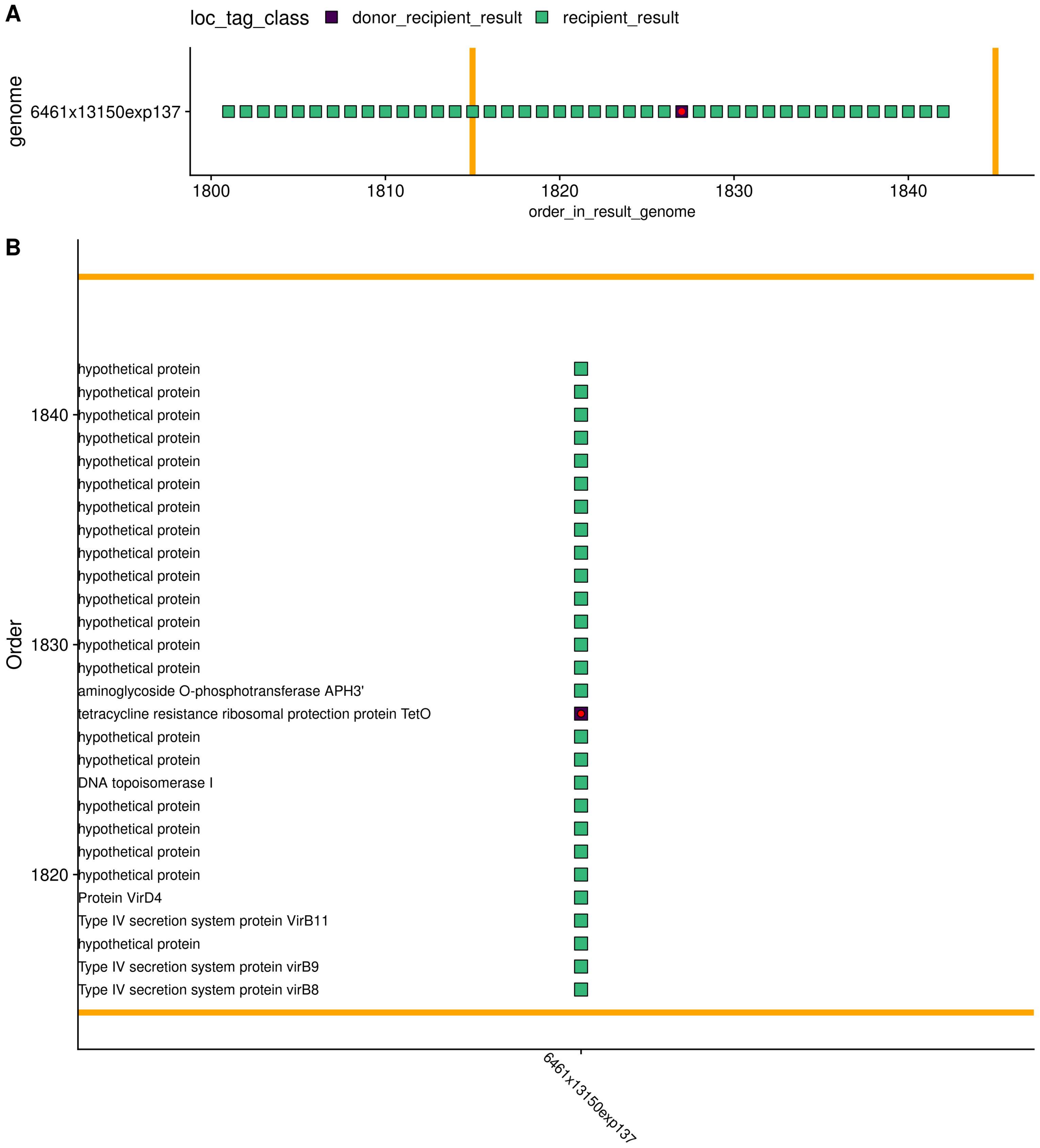

Supplement: Supplementary file 1 [file Data_Sheet_1.PDF]
