# Supplementary material for: Natural Horizontal Gene Transfer of Antimicrobial Resistance Genes in Campylobacter spp. From Turkeys and Swine
Source: Front Microbiol. 2021 Sep 27;12:732969. doi: 10.3389/fmicb.2021.732969 (PMC8504540; doi:10.3389/fmicb.2021.732969)
Supplement: Supplementary file 2 [file Data_Sheet_2.docx]

**Natural horizontal gene transfer of antimicrobial resistance genes in *Campylobacter* spp. from turkeys and swine**

**SUPPLEMENTARY TABLES**

**Table S1: List of media with combination of antibiotics used for the plating of turkey cecal content by room and by necropsy day.** Different combinations were used to recover one (at Day 8) or two parental strains (Day 15 and Day22) as well as a possible newly emerged strain with dual resistance.

| **Necropsy day** | **Room 07**  (control) | **Room 09**  (6461 x 14229-5) | **Room 10**  (6461 x 13150) | **Room 11**  (6461 x 6067) |
| --- | --- | --- | --- | --- |
| **D8** | CAC | CAC  CAC + S | CAC  CAC + K | CAC  CAC + A |
| **D15**  **D22** | CAC | CAC  CAC + S  CAC + K  CAC + G  CAC + S + G | CAC  CAC + S  CAC + K  CAC + T + K | CAC  CAC + S  CAC + A  CAC + S + A |

CAC: CHROMagar^TM^ Campylobacter; A: ampicillin; G: gentamicin; K: kanamycin; S: streptomycin; T: tetracycline.

**Table S2: Minimal inhibitory concentrations (MICs) from CAMPY2 Sensititre® plates (VersaTREK Diagnostics/ThermoFisher, US).** Antimicrobial levels are given in µg/ml. Eight antimicrobials were tested. S: susceptible; R: resistant; NI: no interpretation because guidelines have not been established by the Clinical and Laboratory Standards Institute (CLSI) for the bacteria/drug combination being tested. Testing was conducted by the Veterinary Diagnostic Laboratory at Iowa State University (ISUVDL). Sensitivity testing failed for strain 14229-5, the result provided being “Isolate was unable to grow for sensitivity (two attempts)”.

| Strain | AZI | CIP | CLI | ERY | FFN | GEN | NAL | TET | Test result |
| --- | --- | --- | --- | --- | --- | --- | --- | --- | --- |
| Cj 6631 | NI / ≦0.015 | S / 0.12 | S / 0.06 | S / 0.25 | S / 0.5 | S / ≦0.12 | S / ≦4 | R / 64 | high |
| Cc 13150 | S / 0.06 | R / 16 | S / 0.25 | S / 0.5 | S / 1 | S / 1 | R / >64 | S / 0.25 | low |
| Cj 11601MD | NI / ≦0.015 | R / 4 | S / ≦0.03 | S / 0.12 | S / 0.5 | S / ≦0.12 | S / ≦4 | R / 32 | low |
| Cc 6461 | R / >64 | S / 0.12 | R / 4 | R / >64 | S / 1 | S / 0.5 | S / ≦4 | R / >64 | low |
| Cc 6067 | S / 0.06 | R / 16 | S / 0.25 | S / 1 | S / 1 | S / 0.25 | R / 32 | R / 64 | low |
| Cj JCC | NI / ≦0.015 | S / 0.03 | S / ≦0.03 | S / 0.12 | S / 0.12 | S / ≦0.12 | S / ≦4 | S / 4 | low |
| Cj 14229-5 | - | - | - | - | - | - | - | - | negative |
| 6461x13150_NES *in vivo* | S / 0.03 | R / 16 | S / 0.25 | S / 0.5 | S / 1 | S / 0.5 | R / >64 | R / >64 | low |
| 6461x13150_NES *in vitro* | S / 0.06 | R / 16 | S / 0.25 | S / 0.5 | S / 1 | S / 0.5 | R / >64 | R / >64 | low |

*Cj*: *Campylobacter jejuni*; *Cc*: *Campylobacter coli*; AZI: azithromycin; CIP: ciprofloxacin; CLI: clindamycin; ERY: erythromycin; FFN: florfenicol; GEN: gentamicin; NAL: nalidixic acid; TET: tetracycline.

**Table S3: Unknown mutations (in genes where specific known mutations confer resistance) in *Campylobacter* sp. as identified using the web-based tool ResFinder 4.1** (available at <https://cge.cbs.dtu.dk/services/ResFinder/>) (Zankari et al. 2012). Contigs (FASTA files) from each sample were screened for “Chromosomal point mutations - Unknown mutations” with the default settings of 90% minimum identity and 20% minimum template length.

| **Species**  **and strain** | **Phenotypic**  **resistance** | **Unknown mutation** | **Nucleotide change** | **Amino acid change** |
| --- | --- | --- | --- | --- |
| **Cj 15152A** | AKG | *gyrA* p. N203S | AAT → AGT | N → S |
|  |  | *gyrA* p. R285K | AGG → AAG | R → K |
|  |  | *gyrA* p. A376V | GCA → GTA | A → V |
|  |  | *gyrA* p. Q863* | CAA → TAA | Q → stop |
|  |  | *cmeR* p. G144D | GGT → GAT | G → D |
|  |  | *cmeR* p. P183R | CCT → CGT | P → R |
|  |  | *cmeR* p. S207G | AGC → GGA | S → G |
|  |  | *23S* r. C296G | C → G |  |
|  |  | *23S* r. G298A | G → A |  |
|  |  | *23S* r. G327A | G → A |  |
|  |  | *23S* r. G364C | G → C |  |
|  |  | *23S* r. A554C | A → C |  |
|  |  | *23S* r. T571G | T → G |  |
|  |  | *23S* r. T1601C | T → C |  |
|  |  | *23S* r. A1620G | A → G |  |
|  |  | *23S* r. T1661C | T → C |  |
|  |  | *23S* r. T1987C | T → C |  |
| **Cj 15152B** | AKG | *gyrA* p. N203S | AAT → AGT | N → S |
|  |  | *gyrA* p. R285K | AGG → AAG | R → K |
|  |  | *gyrA* p. A376V | GCA → GTA | A → V |
|  |  | *gyrA* p. Q863* | CAA → TAA | Q → stop |
|  |  | *cmeR* p. G144D | GGT → GAT | G → D |
|  |  | *cmeR* p. P183R | CCT → CGT | P → R |
|  |  | *cmeR* p. S207G | AGC → GGA | S → G |
|  |  | *23S* r. C296G | C → G |  |
|  |  | *23S* r. G298A | G → A |  |
|  |  | *23S* r. G327A | G → A |  |
|  |  | *23S* r. G364C | G → C |  |
|  |  | *23S* r. A554C | A → C |  |
|  |  | *23S* r. T571G | T → G |  |
|  |  | *23S* r. T2854C | T → C |  |
| **Cc 13150** | AKQ | *23S* r. C296G | C → G |  |
|  |  | *23S* r. G364C | G → C |  |
|  |  | *23S* r. G402A | G → A |  |
|  |  | *23S* r. T416G | T → G |  |
|  |  | *23S* r. C418T | C → T |  |
|  |  | *23S* r. A554C | A → C |  |
|  |  | *23S* r. T571G | T → G |  |
|  |  | *23S* r. A1730C | A → C |  |
|  |  | *23S* r. T1735C | T → C |  |
|  |  | *23S* r. T1752C | T → C |  |
|  |  | *23S* r. A1759G | A → G |  |
|  |  | *23S* r. G1761A | G → A |  |
|  |  | *23S* r. C2113T | C → T |  |
| **Cj 14398-5** | AKG | *gyrA* p. N203S | AAT → AGT | N → S |
|  |  | *gyrA* p. R285K | AGG → AAG | R → K |
|  |  | *gyrA* p. A376V | GCA → GTA | A → V |
|  |  | *gyrA* p. Q863* | CAA → TAA | Q → stop |
|  |  | *cmeR* p. G144D | GGT → GAT | G → D |
|  |  | *cmeR* p. P183R | CCT → CGT | P → R |
|  |  | *cmeR* p. S207G | AGC → GGA | S → G |
|  |  | *23S* r. C296G | C → G |  |
|  |  | *23S* r. G298A | G → A |  |
|  |  | *23S* r. G327A | G → A |  |
|  |  | *23S* r. G364C | G → C |  |
|  |  | *23S* r. A554C | A → C |  |
|  |  | *23S* r. T571G | T → G |  |
|  |  | *23S* r. T1752C | T → C |  |
| **Cj 11601MD** | AKQT | *gyrA* p. N203S | AAT → AGT | N → S |
|  |  | *gyrA* p. R285K | AGG → AAG | R → K |
|  |  | *gyrA* p. Q863* | CAA → TAA | Q → stop |
| **Cc 6461** | TSE | *23S* r. C240T | C → T |  |
|  |  | *23S* r. C296G | C → G |  |
|  |  | *23S* r. C264C | G → C |  |
|  |  | *23S* r. G402A | G → A |  |
|  |  | *23S* r. T416G | T → G |  |
|  |  | *23S* r. C418T | C → T |  |
|  |  | *23S* r. G1579A | G → A |  |
|  |  | *23S* r. A1730C | A → C |  |
|  |  | *23S* r. T1735C | T → C |  |
|  |  | *23S* r. T1752C | T → C |  |
|  |  | *23S* r. A1759G | A → G |  |
|  |  | *23S* r. G1761A | G → A |  |
|  |  | *23S* r. C2113T | C → T |  |
| **Cj 14229-5** | AKGT | *gyrA* p. N203S | AAT → AGT | N → S |
|  |  | *gyrA* p. R285K | AGG → AAG | R → K |
|  |  | *gyrA* p. Q863* | CAA → TAA | Q → stop |
|  |  | *cmeR* p. G86S | GGC → AGC | G → S |
|  |  | *cmeR* p. S207G | AGC → GGA | S → G |
| **Cj JCC** | T | *gyrA* p. S22G | AGT → GGT | S → G |
|  |  | *gyrA* p. N203S | AAT → AGT | N → S |
|  |  | *gyrA* p. R285K | AGG → AAG | R → K |
|  |  | *gyrA* p. V720I | GTA → ATA | V → I |
|  |  | *gyrA* p. Q863* | CAA → TAA | Q → stop |
|  |  | *cmeR* p. T6I | ACA → ATA | T → I |
|  |  | *cmeR* p. G144D | GGT → GAT | G → D |
|  |  | *cmeR* p. P183R | CCT → CGT | P → R |
|  |  | *cmeR* p. S207G | AGC → GGA | S → G |
|  |  | *23S* r. C296G | C → G |  |
|  |  | *23S* r. G298A | G → A |  |
|  |  | *23S* r. G327A | G → A |  |
|  |  | *23S* r. G364C | G → C |  |
|  |  | *23S* r. A554C | A → C |  |
|  |  | *23S* r. T571G | T → G |  |
|  |  | *23S* r. A1496G | A → G |  |
|  |  | *23S* r. T1752C | T → C |  |
| **Cj 6535** | ATS | *gyrA* p. N203S | AAT → AGT | N → S |
|  |  | *gyrA* p. R285K | AGG → AAG | R → K |
|  |  | *gyrA* p. H339Y | CAT → TAC | H → Y |
|  |  | *gyrA* p. E393K | GAA → AAA | E → K |
|  |  | *cmeR* p. G53S | GGT → AGT | G → S |
|  |  | *cmeR* p. E84K | GAA → AAA | E → K |
|  |  | *cmeR* p. G86S | GGC → AGT | G → S |
|  |  | *cmeR* p. I100L | ATA → TTG | I → L |
|  |  | *cmeR* p. M154I | ATG → ATA | M → I |
|  |  | *cmeR* p. K160R | ATG → ATA | K → R |
|  |  | *cmeR* p. T167S | ACT → TCT | T → S |
|  |  | *cmeR* p. A182T | GCT → ACT | A → T |
| **Cj 6631** | ATS | *gyrA* p. N203S | AAT → AGT | N → S |
|  |  | *gyrA* p. R285K | AGG → AAG | R → K |
|  |  | *gyrA* p. H339Y | CAT → TAC | H → Y |
|  |  | *gyrA* p. E393K | GAA → AAA | E → K |
|  |  | *cmeR* p. G53S | GGT → AGT | G → S |
|  |  | *cmeR* p. E84K | GAA → AAA | E → K |
|  |  | *cmeR* p. G86S | GGC → AGT | G → S |
|  |  | *cmeR* p. I100L | ATA → TTG | I → L |
|  |  | *cmeR* p. M154I | ATG → ATA | M → I |
|  |  | *cmeR* p. K160R | ATG → ATA | K → R |
|  |  | *cmeR* p. T167S | ACT → TCT | T → S |
|  |  | *cmeR* p. A182T | GCT → ACT | A → T |
| **Cc 6067** | AQT | *23S* r. C296G | C → G |  |
|  |  | *23S* r. G364C | G → C |  |
|  |  | *23S* r. G402A | G → A |  |
|  |  | *23S* r. T416G | T → G |  |
|  |  | *23S* r. C418T | C → T |  |
|  |  | *23S* r. A554C | A → C |  |
|  |  | *23S* r. T571G | T → G |  |
|  |  | *23S* r. A1730C | A → C |  |
|  |  | *23S* r. T1735C | T → C |  |
|  |  | *23S* r. T1752C | T → C |  |
|  |  | *23S* r. A1759G | A → G |  |
|  |  | *23S* r. G1761A | G → A |  |
|  |  | *23S* r. C2113T | C → T |  |

Cc: Campylobacter coli. Cj: Campylobacter jejuni. A: ampicillin/carbenicillin; E: erythromycin; G: gentamicin; K: kanamycin; Q: nalidixic acid and ciprofloxacin; S: streptomycin; T: tetracycline.

**Table S4: Detailed horizontal gene transfers observe in the newly emerged strains (NES).** For each *in vitro* transfer experiment, three genomes (from two parental and one NES strain) were included in the pangenome analysis using Roary (Page et al. 2015). The stringency for assigning coding sequences to a gene was set to 99% identity. The tool gifrop (v0.0.6; <https://github.com/Jtrachsel/gifrop>) was used to help identify transfer of genes or consecutive blocks of genes – hereafter referred to as a ‘genomic island’ ­– absent in the recipient strain but present in the NES. The workflow used to complete this analysis is available at <https://github.com/Jtrachsel/Campy_HGT>. Genes flagged as “resistance genes” are highlighted in yellow; genes flagged as “virulence associated” are highlighted in blue.

**Experiment co-culture Cj JCC and Cc 13150:**

| Gene | Annotation | Order within Fragment |
| --- | --- | --- |
| group_1624 | hypothetical protein | 22 |
| group_1625 | hypothetical protein | 23 |

*No resistance gene transfer detected.*

**Experiment co-culture Cj 6631 and Cc 13150:**

| Gene | Annotation | Order within Fragment |
| --- | --- | --- |
| trpC | Indole-3-glycerol phosphate synthase | 617 |
| group_1640 | lipoprotein | 618 |
| group_1639 | hypothetical protein | 619 |
| group_1638 | methyltransferase domain-containing protein | 620 |
| group_1637 | exporting protein | 621 |

*No resistance gene transfer detected.*

**Experiment co-culture Cj 14229-5 and Cc 6461:**

| Gene | Annotation | Order within Fragment |
| --- | --- | --- |
| group_365 | hypothetical protein | 200 |
| group_364 | aminoglycoside O-phosphotransferase APH(2'')-If | 201 |
| group_363 | IS200/IS605 family transposase IS605 | 202 |
| group_366 | hypothetical protein | 1526 |
| group_367 | IS1595 family transposase ISAcsp6 | 1527 |
| group_368 | hypothetical protein | 1528 |
| pseD_1 | protein PseD | 1531 |

*aph(2”)-If resistance gene transfer detected. pseD is a gene associated with virulence.*

**Experiment co-culture Cc 6067 and Cc 6461:**

| Gene | Annotation | Order within Fragment |
| --- | --- | --- |
| cdtB_2 | cytolethal distending toxin B | 1305 |
| cdtB_1 | cytolethal distending toxin B | 1306 |
| pgtP_3 | Phosphoglycerate transporter protein | 1308 |
| pgtP_2 | Phosphoglycerate transporter protein | 1309 |
| pgtP_1 | Phosphoglycerate transporter protein | 1310 |
| group_176 | acetyltransferase | 1313 |
| group_177 | OXA-61 family class D beta-lactamase OXA-594 | 1317 |
| modC | molybdenum ABC transporter ATP-binding protein | 1318 |
| cdtB_3 | cytolethal distending toxin B | 2504 |

*blaOXA resistance gene transfer detected.*

**Experiment co-culture Cc 6461 and Cc 13150:**

| Gene | Annotation | Order within Fragment |
| --- | --- | --- |
| group_1104 | hypothetical protein | 124 |
| group_1105 | hypothetical protein | 126 |
| group_1106 | carbon-nitrogen hydrolase | 127 |
| group_1119 | multidrug resistance protein | 160 |
| ppi | peptidyl-prolyl cis-trans isomerase | 163 |
| group_1121 | integral membrane protein | 164 |
| ldh | L-lactate dehydrogenase | 165 |
| group_1123 | integral membrane protein | 166 |
| group_1124 | integral membrane protein | 167 |
| group_1125 | hypothetical protein | 168 |
| group_1164 | lipoprotein | 263 |
| group_1165 | hypothetical protein | 264 |
| folC | bifunctional folylpolyglutamate synthase/dihydrofolate synthase | 265 |
| group_1103 | exporting protein | 1638 |
| group_1102 | laccase domain-containing protein | 1643 |
| ribA_2 | riboflavin synthase subunit alpha | 1644 |
| group_1100 | periplasmic protein | 1645 |
| groES | co-chaperonin GroES | 1646 |
| group_1088 | hypothetical protein | 1671 |
| group_1087 | hypothetical protein | 1672 |
| group_1086 | two-component sensor histidine kinase | 1673 |
| htrA | serine protease | 1677 |
| group_1084 | HAD-superfamily hydrolase | 1684 |
| group_1083 | peptidase M23 family protein | 1686 |
| group_908 | radical SAM domain-containing protein | 2163 |
| group_907 | iron-binding protein | 2167 |
| group_906 | iron-binding protein | 2168 |
| group_905 | iron-binding protein | 2169 |
| group_904 | hypothetical protein | 2174 |
| sdaA | L-serine dehydratase | 2177 |
| sdaC | amino acid transporter | 2178 |
| fusA_1 | tetracycline resistance ribosomal protection protein Tet(O) | 2180 |
| group_901 | hypothetical protein | 2181 |
| group_900 | hypothetical protein | 2185 |
| group_899 | periplasmic protein | 2186 |
| fhaC_1 | Filamentous hemagglutinin transporter protein FhaC | 2190 |
| group_897 | hypothetical protein | 2191 |
| group_896 | hypothetical protein | 2196 |
| group_895 | hypothetical protein | 2197 |
| group_894 | hypothetical protein | 2198 |
| aroC | chorismate synthase | 2199 |
| rnc | ribonuclease III | 2200 |

*tet(O) resistance gene transfer detected.*

**Experiment *in vivo* dual inoculation Cc 6461 and Cc 13150:**

| Gene | Annotation | Order within Fragment |
| --- | --- | --- |
| group_1024 | motility protein | 549 |
| flaA | flagellin A | 550 |
| flaB | flagellin B | 551 |
| group_1023 | motility protein | 1633 |
| group_29 | hypothetical protein | 1634 |
| group_1022 | periplasmic protein | 1635 |
| group_1021 | tRNA N6-adenosine threonylcarbamoyltransferase | 1636 |
| group_896 | hypothetical protein | 2458 |
| group_895 | periplasmic protein | 2459 |
| group_894 | Filamentous hemagglutinin transporter protein FhaC | 2462 |

*No resistance gene transfer detected in the pangenome analysis. However, present tet(O) gene was replaced by another version of a tet(O) gene from the donor strain following homologous recombination.*

*Several genes are associated with virulence (flaA, flab, pseE/maf5, maf4, pseD/maf2).*
